# Estimating Reaction Rate Constants from Impedance Spectra: Combining Microkinetic Modeling and Experiments of the Oxygen Evolution Reaction

**DOI:** 10.1021/acs.jpcc.6c00968

**Published:** 2026-04-13

**Authors:** B. F. H. van den Boorn, F. Vandeputte, M. van Berkel, C. Mempin, D. Sarkar, J. Lataire, G. Vandersteen, A. Bieberle-Hütter

**Affiliations:** † Electrochemical Materials and Interfaces, 55956 DIFFER—Dutch Institute for Fundamental Energy Research, Eindhoven 5612 AE, The Netherlands; ‡ Electrical Engineering, Eindhoven University of Technology, Eindhoven 5612 AE, The Netherlands; § ELEC, 70493 Vrije Universiteit Brussel, Brussels 1050, Belgium; ∥ Energy Systems and Control, DIFFER—Dutch Institute for Fundamental Energy Research, Eindhoven 5612 AE, The Netherlands

## Abstract

Knowledge of the reaction rate constants can be vital in understanding electrochemical reaction mechanisms and their rate-determining processes. Although first-principles methods, such as density functional theory (DFT), provide valuable insight into reaction free energies and rate constants, they commonly use idealized assumptions. As a different approach, this study demonstrates the estimation of rate constants from electrochemical data. Specifically, this study estimates rate constants from electrochemical impedance spectroscopy (EIS) data of the oxygen evolution reaction (OER) with a hematite Fe_2_O_3_ anode often used in photoelectrochemical cells. Unlike the common approach of equivalent circuit fitting with resistances and capacitances, the electrochemistry of the OER in this work is represented by a microkinetic model and physicochemical quantities. The estimated rate constants directly correspond to the OER reaction steps, and a single set of rate constants is obtained that is optimized for multiple potentials simultaneously. The estimation is conducted using maximum likelihood estimation. The effectiveness of the estimation method is shown using synthetic measurements first; intermediate species coverages are simulated as well. Then, the rate constants are estimated directly from experimental EIS measurements. Though accuracy is currently limited due to the model that does not account for all processes at the interface, such as, for example, diffusive transport, the estimated rate constants do represent the experimental interface and are perfectly suited for kinetic analysis and systematic parameter studies of the electrochemical system. Additionally, this approach enables analyses of differences between electrode materials, validation of models, and prediction of electrochemical data of different material systems, which is time-consuming and costly to obtain experimentally. This research demonstrates how combining potential- and frequency-dependent EIS experiments with microkinetic modeling enables the estimation of reaction rates and intermediate species coverages, aiding in identifying reaction mechanisms directly from experiments.

## Graphical abstract

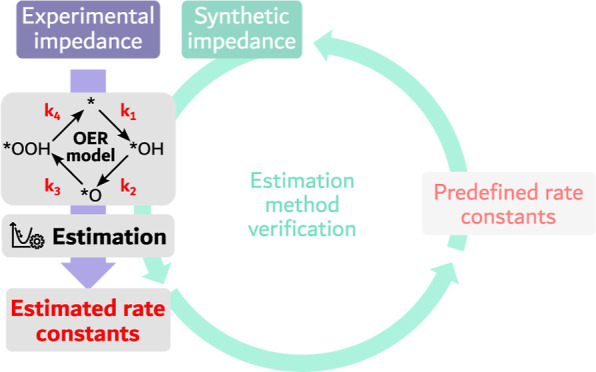

## 1 Introduction

Photoelectrochemical (PEC) water splitting is a promising method for the production of green hydrogen utilizing solar energy in a single integrated device. However, the current efficiency of PEC water splitting is insufficient to reach market competitiveness with alternative solar-to-green-hydrogen methods, such as coupled photovoltaic-electrolysis.^1^ The efficiency of water splitting is primarily limited by the oxygen evolution reaction (OER), and advances in efficiency can be realized through the discovery of new electrode materials with favorable kinetic properties for the OER. A theoretically informed comparison of prospective materials can be facilitated by modeling the kinetics of the OER for different materials.

Modeling reaction kinetics is complex because of the interplay of different chemical processes contributing across time and length scales; for example, atomistic scale properties affect reaction kinetics, even at the continuum level. Therefore, accurate modeling of reaction kinetics requires connecting advanced computational methods at different scales; hence, a multiscale approach is needed.^2–4^ Recent studies have modeled the OER using various approaches on different scales.^3^ So far, most studies focus on the atomistic scale, using Density Functional Theory (DFT) calculations to determine the free energies of the reaction steps, the reaction rate constants, and the potential-determining step for the OER.^2,5–12^ Piqué et al. (2020) utilized these free energies for enhanced OER catalyst design.^13^ Recent molecular-scale studies have investigated the reaction kinetics of the hematite-liquid water interface using ab initio molecular dynamics (MD)^14^ and force field MD methods.^15^ The development of these force field models has recently been accelerated by machine learning approaches.^16–18^ Furthermore, mesoscale studies employ Monte Carlo techniques to estimate the predominant reaction paths of a given mechanism;^3,19^ However, these techniques have not been widely used for OER and have been described as an “open challenge”.^3^ Recent continuum scale studies have investigated electrochemical charge transfer at the interface using microkinetic modeling.^2,3,10,20,21^ Electrochemical data, such as current–voltage curves and electrochemical impedance spectra (EIS), have been simulated directly from electrochemical models.^3,20^ Finally, system-scale studies have developed multiphysics models that incorporate interface and electrolyte physics.^22–24^

Crucial for analyzing the reaction kinetics are the reaction rate constants, which can indicate the rate-determining step of the mechanism. These rate constants cannot be measured experimentally and instead are commonly modeled using DFT and MD by calculating free energies.^10,20^ However, these methods have several limitations: For example, DFT simulates a solid–gas interface, whereas the OER for water splitting occurs at a solid–liquid interface. Additionally, DFT uses idealized configurations at temperatures near absolute zero and suffers from high computational costs. Furthermore, MD methods are sensitive to variations in the chosen force field model and are limited by high computational cost.^3,25^ Hence, it is highly desired to determine the reaction rate constants without relying on these potentially inaccurate atomistic approaches. The determination of the reaction rate constants can furthermore be used to predict data, such as current–voltage (*I*–*V*) characteristics and EIS data, of different material systems.

Therefore, in this work, we estimate the reaction rate constants of the OER directly from EIS data. Hematite α-Fe_2_O_3_ is chosen as the anode material. A previously developed microkinetic model is used for the estimation.^10^ It represents the four-step OER mechanism proposed by Rossmeisl and Nørskov (2007).^7^ The estimation is carried out by iterative minimization of the difference between the EIS data and the microkinetic model. Garcia-Osorio et al. (2017) previously estimated OER rate constants using an iterative procedure with a sum-of-squares error fitness function on boron-doped diamond, SnO_2_–Sb, and PbO_2_.^26^ In contrast, our study employs maximum likelihood estimation, using the measurement uncertainty of multiple measurements as a weighting for an accurate estimation. Furthermore, we obtain a single set of rate constants optimized for impedance measurements at multiple frequencies and potentials simultaneously. This study demonstrates that our methodology is effective, providing an accurate estimation of the rate constants from synthetic EIS measurements. When the rate constants are estimated directly from experimental EIS measurements, their accuracy is currently limited because the model does not account for all processes at the interface, such as, for example, diffusive transport. Furthermore, it is shown that EIS measurements at low frequency and high potential are favorable for the estimation of the rate constants by analyzing the Cramer-Rao lower bound (CRLB). When included, the microkinetic OER model shows greater sensitivity to transport-related parameters than to the rate constants. This underscores the importance of identifying transport processes in experimental EIS measurements prior to estimating rate constants. Finally, our research demonstrates that the way EIS data is processed by the potentiostat software can affect the accuracy of the rate constant estimation.

The remainder of the paper is structured as follows. Section 2 describes the modeled OER mechanism and provides a theoretical background of the reaction rate constants. Section 3 covers the various methods used in the paper, starting with the theoretical methods, which include the microkinetic model, the equivalent circuit, and the estimation method. A more extensive description of the latter is given in the companion paper.^27^ Section 4 presents the results of the rate constant estimation for the simulated and experimental data. Finally, a conclusion is given in Section 5.

## 2 Theory

### 2.1 Oxygen Evolution Reaction Mechanism

The oxygen evolution mechanism used in this work is the well-known reaction mechanism in an alkaline environment, as proposed by Rossmeisl and Nørskov (2007).^7^ This mechanism is chosen because of its relative simplicity, consisting of only four reaction steps. These four steps, together with the oxygen desorption step, are given as follows.

1 $${\;}^{*}+{OH}^{-}+h^{+}\overset{K_{f1}}{\underset{K_{b1}}{⇌}}{OH}^{*}$$

2 $${OH}^{*}+{OH}^{-}+h^{+}\overset{K_{f2}}{\underset{K_{b2}}{⇌}}O^{*}+H_{2}O$$

3 $$O^{*}+{OH}^{-}+h^{+}\overset{K_{f3}}{\underset{K_{b3}}{⇌}}{OOH}^{*}$$

4 $${OOH}^{*}+{OH}^{-}+h^{+}\overset{K_{f4}}{\underset{K_{b4}}{⇌}}{O_{2}}^{*}+H_{2}O$$

5 $${O_{2}}^{*}\overset{K_{f5}}{\to }O_{2}+{\;}^{*}$$

where X* indicates an adsorbed species X and * (separately) indicates a free surface site. The forward and backward reaction rates for each step *i*, *i* ∈ {1, ..., 5}, are denoted *K*_f*i*_ and *K*_b*i*_ [cm s^–1^], respectively.

### 2.2 Reaction Rate Constants

The potential dependent reaction rates *K*_f*i*_ and *K*_b*i*_ in eqs 1–4 can be expressed in the potential independent reaction rate constants *k*_f*i*_ and *k*_b*i*_ [cm^4^ s^–1^] as

6 $$K_{fi}=k_{fi}p_{s}\left(u_{app}\right)$$

7 $$K_{bi}=k_{bi}N_{v}$$

where *p*_s_(*u*_app_) [cm^–3^] is the density of holes available on the semiconductor surface and *N*_v_ [cm^–3^] is the effective density of states on the semiconductor surface. The hole density depends on the applied potential, *u*_app_ [V], according to^28^

8 $$p_{s}=p_{s}^{0}\;\exp\left(\frac{u_{app}-jR_{s}}{k_{B}T}\right)$$

where *p*_s_^0^ [cm^–3^] is the hole density at the surface in the dark in equilibrium, the current density is *j* [A cm^–2^], series resistance is *R*_s_ [Ω], Boltzmann constant is *k*_B_ [eV K^–1^], and temperature *T* = 298 K. Note that this description of the hole density represents only dark conditions. This description is used in this work as a simplified case to illustrate how to estimate the rate constants. In parallel, we are working on a more complex model that simulates the effect of illumination and also includes a more detailed model of the charge carrier dynamics; this model can be used in the future for the estimation of the rate constants. Furthermore, the description of the reaction rates in eqs 6 and 7 assumes that charge transfer occurs exclusively through the valence band.^10^ Although charge transfer can occur through both the valence and conduction bands, for an anodic reaction in an n-type semiconductor, such as hematite used in this work, it occurs primarily through the valence band.^28^

The rate constants can be theoretically derived from Gibbs free energies, expanding the constants in a pre-exponential and an exponential term^28^

9 $$k_{fi}\left(\Delta G_{i}\right)=k_{\max}\;\exp\left(-\frac{{\left(E_{v}-E_{F,redox,i}^{0}-\lambda \right)}^{2}}{4k_{B}T\lambda }\right)$$

10 $$k_{bi}\left(\Delta G_{i}\right)=k_{\max}\;\exp\left(-\frac{{\left(E_{v}-E_{F,redox,i}^{0}+\lambda \right)}^{2}}{4k_{B}T\lambda }\right)$$

The exponential term follows from the distribution of empty energy states of the redox system, as described in the Gerisher model.^28^ The approximation assumes that charge transfer occurs mainly within 1 *k*_B_ *T* of the edge of the valence band.^28^ The exponential term includes the valence band energy level, *E*_v_ [eV], the Fermi level of each of the reaction steps $iE_{F,redox,i}^{0}\left(\Delta G_{i}\right)$ [eV], and the solvent reorganization energy λ [eV]. *E*_F,redox,*i*_^0^(Δ*G*_*i*_) is related to the Gibbs free energy change for each reaction as

11 $$E_{F,redox,i}^{0}=\frac{\Delta G_{i}}{nF_{a}}$$

where the number of electrons transferred per step, *n* = 1 and the Faraday constant is *F*_a_. The pre-exponential term in eqs 9 and 10 is the maximum rate for valence band hole transfer, *k*_max_ = 1.76 × 10^–16^ cm^4^ s^–1^, which is assumed constant.^28,29^

## 3 Methods

### 3.1 OER Model

The microkinetic OER model used for the estimation in this study was developed in an earlier study, and we refer the reader to that publication for a detailed description of the model.^10^ In this section, the key governing model equations are introduced, and an expression is derived for the impedance over the semiconductor-electrolyte interface (SEI). First, the mass balance equations are derived from the reaction steps in Section 2.1. Second, these equations are linearized around their operating points, a standard procedure for studying EIS, and the expression for the impedance is obtained. Third, the steady-state coverage expressions are derived from the mass balance equations in the steady state. These coverage expressions are functions of the rate constants and are substituted in the model.

#### 3.1.1 Mass Balance Equations

The OER mechanism in eqs 1–4 can be reformulated as mass balance equations, describing the rate of change of each of the coverage species. Following the analysis in Vandeputte et al. (2025), a reduced model with only the coverages of the three species OH, O, and OOH adequately describes the dynamics in the relevant frequency range (100 mHz-300 kHz), omitting O_2_.^27^ Accordingly, the mass balance equations are given as follows

12 $$\left[\begin{array}{l} {\mathrm{\theta ̇}}_{1}\\ {\mathrm{\theta ̇}}_{2}\\ {\mathrm{\theta ̇}}_{3} \end{array}\right]=\left[\begin{array}{l} {\mathrm{K̅}}_{f1}\theta_{*}-{\mathrm{K̅}}_{b1}\theta_{1}-{\mathrm{K̅}}_{f2}\theta_{1}+{\mathrm{K̅}}_{b2}\theta_{2}\\ {\mathrm{K̅}}_{f2}\theta_{1}-{\mathrm{K̅}}_{b2}\theta_{2}-{\mathrm{K̅}}_{f3}\theta_{2}+{\mathrm{K̅}}_{b3}\theta_{3}\\ {\mathrm{K̅}}_{f3}\theta_{2}-{\mathrm{K̅}}_{b3}\theta_{3}-{\mathrm{K̅}}_{f4}\theta_{3} \end{array}\right]$$

where the species coverages are denoted as θ_*j*_ with *j* (1, 2, 3) ≔ (OH, O, OOH), the time evolution of the coverages is given as ${\mathrm{\theta ̇}}_{j}$ , and θ_*_ = 1 – θ_1_ – θ_2_ – θ_3_. Furthermore, the mole fraction of hydroxide ions *x*_OH_ and water are included as follows

13 $${\mathrm{K̅}}_{fi}=K_{fi}x_{OH},\text{for}\;i=\left(1,2,3\right)$$

14 $${\mathrm{K̅}}_{bi}=\{\begin{array}{ll} K_{bi} & \text{for}\;i=\left(1,3\right)\\ K_{bi}x_{H_{2}O} & \text{for}\;i=2 \end{array}$$

The current density is obtained from the reaction rates and species coverages, according to

15 $$\begin{array}{l} j_{f}=eN_{0}({\mathrm{K̅}}_{f1}\left(u_{app}\right)\theta_{*}+{\mathrm{K̅}}_{f2}\left(u_{app}\right)\theta_{1}\\ +{\mathrm{K̅}}_{f3}\left(u_{app}\right)\theta_{2}+{\mathrm{K̅}}_{f4}\left(u_{app}\right)\theta_{3}) \end{array}$$

16 $$j_{b}=eN_{0}\left({\mathrm{K̅}}_{b1}\theta_{1}+{\mathrm{K̅}}_{b2}\theta_{2}+{\mathrm{K̅}}_{b3}\theta_{3}\right)$$

17 $$j=j_{f}-j_{b}$$

where *N*_0_ is the density of surface adsorption sites.

#### 3.1.2 Linearization and Impedance

EIS analyzes the dynamics of a system by applying an excitation signal, *ũ*(*t*), around a steady-state potential, *u* ^eq^. Given a sufficiently small potential excitation, the dynamics are assumed to be linear around the steady-state operating point. Accordingly, the model is linearized around *u* ^eq^, using the following expressions for the coverage, applied potential, and current density response

18 $$\theta \left(t\right)=\theta^{eq}+\mathrm{\theta ̃}\left(t\right)$$

19 $$u_{app}\left(t\right)=u^{eq}+ũ\left(t\right)$$

20 $$j\left(t\right)=j^{eq}+\mathrm{j̃}\left(t\right)$$

where θ^eq^ and *j* ^eq^ describe the steady-state coverages and steady-state current density, respectively, and θ̃(*t*) and *j̃*(*t*) are the excitation signals of the coverages and current density, respectively.

The linearized system is represented in the form of a state-space system^30^

21 $$\dot{\tilde{\theta }}\left(t\right)=A\mathrm{\theta ̃}\left(t\right)+Bũ\left(t\right)$$

22 j̃(t)=Cθ̃(t)+Dũ(t)

where $\dot{\tilde{\theta }}$ represents the time evolution of the coverages under excitation, and the matrix terms are determined as follows: $A={\frac{\partial \mathrm{\theta ̇}}{\partial \theta }|}_{\theta^{eq},u^{eq}}$ , $B={\frac{\partial \mathrm{\theta ̇}}{{\partial u}_{app}}|}_{\theta^{eq},u^{eq}}$ , $C={\frac{\partial j}{\partial \theta }|}_{\theta^{eq},u^{eq}}$ , and $D={\frac{\partial j}{\partial u_{app}}|}_{\theta^{eq},u^{eq}}$ .

The periodic potential excitation and current density are typically converted to the frequency domain, using the Fourier transform U(ω)=F{ũ(t)} and J(ω)=F{j̃(t)} , with ω the angular frequency. The ratio of the frequency domain current density and potential represents the admittance over the SEI and is derived from the linearized system and equals^30^

23 $$G\left(s\right)=\frac{J\left(s\right)}{U\left(s\right)}=C{\left(sI-A\right)}^{-1}B+D$$

where *I* is the identity matrix, the Laplace variable *s* = *i*ω, and *i* is the imaginary unit. The impedance of the system, *Z*_SEI_(*s*), is the reciprocal of the admittance and is given as

24 $$Z_{SEI}\left(s\right)=G^{-1}\left(s\right)$$

#### 3.1.3 Exclusion of Steady-State Coverage Parameters

The SEI impedance *Z*_SEI_(*i*ω) can be modeled by using steady-state potential and steady-state coverage of surface species. However, the latter cannot be accurately measured using experimental techniques. Therefore, the surface coverages are eliminated by solving the mass balance equations (eq 12) in steady-state, ${\mathrm{\theta ̇}}^{eq}=0$ , and expressing the coverages in the rate constants

25 $$\theta_{1}^{eq}\left(k,u^{eq}\right)=\theta_{den}^{-1}\left({\mathrm{K̅}}_{f1}\left({\mathrm{K̅}}_{b2}\left({\mathrm{K̅}}_{b3}+{\mathrm{K̅}}_{f4}\right)+{\mathrm{K̅}}_{f3}{\mathrm{K̅}}_{f4}\right)\right)$$

26 $$\theta_{2}^{eq}\left(k,u^{eq}\right)=\theta_{den}^{-1}\left({\mathrm{K̅}}_{f1}{\mathrm{K̅}}_{f2}\left({\mathrm{K̅}}_{b3}+{\mathrm{K̅}}_{f4}\right)\right)$$

27 $$\theta_{3}^{eq}\left(k,u^{eq}\right)=\theta_{den}^{-1}\left({\mathrm{K̅}}_{f1}{\mathrm{K̅}}_{f2}{\mathrm{K̅}}_{f3}\right)$$

28 $$\theta_{4}^{eq}\left(k,u^{eq}\right)=0$$

where θ_4_ ≔ θ_O_2__ and the denominator term

29 $$\begin{array}{ll} \theta_{den}\left(k\right) & ={\mathrm{K̅}}_{f1}{\mathrm{K̅}}_{f2}\left({\mathrm{K̅}}_{f3}+{\mathrm{K̅}}_{b3}+{\mathrm{K̅}}_{f4}\right)\\ & +{\mathrm{K̅}}_{f1}{\mathrm{K̅}}_{b2}\left({\mathrm{K̅}}_{b3}+{\mathrm{K̅}}_{f4}\right)+{\mathrm{K̅}}_{f1}{\mathrm{K̅}}_{f3}{\mathrm{K̅}}_{f4}\\ & +{\mathrm{K̅}}_{b1}{\mathrm{K̅}}_{b2}\left({\mathrm{K̅}}_{b3}+{\mathrm{K̅}}_{f4}\right)+{\mathrm{K̅}}_{b1}{\mathrm{K̅}}_{f3}{\mathrm{K̅}}_{f4}\\ & +{\mathrm{K̅}}_{f2}{\mathrm{K̅}}_{f3}{\mathrm{K̅}}_{f4} \end{array}$$

Here and in the remainder of this work, the reaction rate constants are collectively referred to as **k** = [*k*_f1_, *k*_b1_, *k*_f2_, *k*_b2_, *k*_f3_, *k*_b3_, *k*_f4_].

### 3.2 Equivalent Circuit Extension

The SEI impedance *Z*_SEI_(*i*ω) obtained from eq 24 represents the charge transfer processes at the SEI. This SEI impedance is only part of the overall impedance of the electrolysis cell, *Z*_cell_(*i*ω), which is measured in experiments between the working electrode and the reference electrode (RE) (see Figure 1). Hence, in order to compare simulated impedance with experimental impedance, the simulated *Z*_SEI_(*i*ω) also needs to be extended to include the additional contributions in *Z*_cell_(*i*ω) from the experimental measurements. Therefore, to relate the experimental *Z*_cell_(*i*ω) and the simulated *Z*_SEI_(*i*ω), the impedance model from eq 24 was placed within a commonly used equivalent circuit model from the literature for this type of system (Figure 1).^31,32^

**Figure 1:**
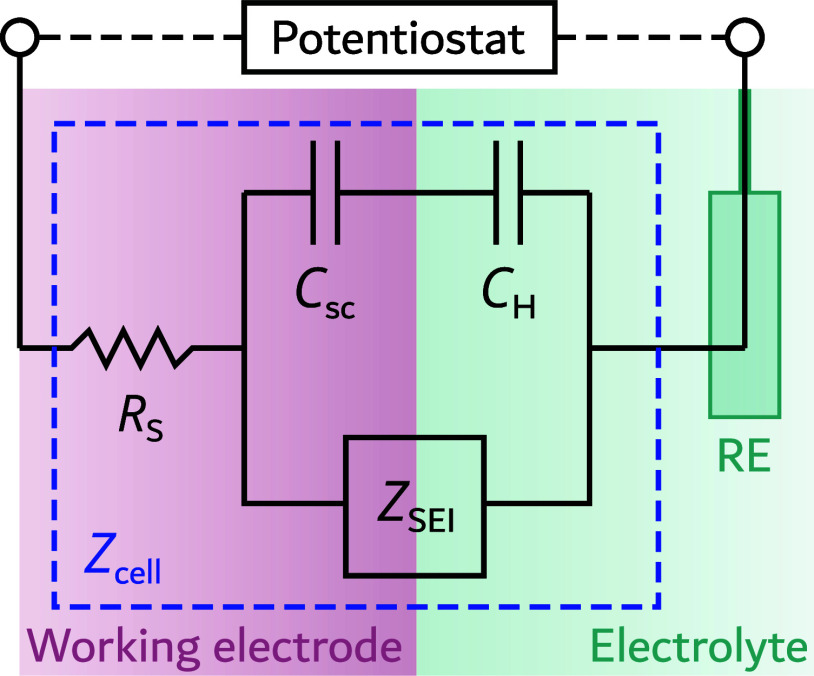
Equivalent circuit representing the electrolysis cell impedance, *Z*_cell_, as well as the RE. The space-charge capacitance, *C*_sc_, and Helmholtz capacitance, *C*_H_, are in parallel to the SEI impedance, *Z*_SEI_, representing the OER charge transfer; *R*_s_ is the series resistance.

The following components are present next to *Z*_SEI_(*i*ω): Firstly, the series resistance *R*_s_ combines the resistances of the back contact and the solution. These two resistances cannot be distinguished in a measurement and are therefore typically combined in a single term.^32,33^ Secondly, the space-charge capacitance *C*_sc_ follows from the space-charge layer, or depletion layer, formed in the semiconductor as the chemical potential of its electrons equilibrates with the chemical potential of reactants in the electrolyte. *C*_sc_ is described by the Mott-Schottky equation, which is given as follows^31^

30 $${\left(\frac{1}{C_{sc}\left(u^{eq}\right)}\right)}^{2}=\frac{2}{\epsilon_{r}\epsilon_{0}eN_{D}}\left(u^{eq}-\frac{k_{B}T}{e}\right)$$

where ϵ_0_ is the permittivity of free space, ϵ_r_ is the relative permittivity of the semiconductor, *e* is the electronic charge, and *N*_D_ is the doping density. Finally, Helmholtz capacitance *C*_H_ is formed at the interface between the semiconductor and the electrolyte. Here, two layers are formed, the inner and outer Helmholtz layers. The inner Helmholtz layer is a monolayer of surface-adsorbed solvent ions of opposite polarity to the semiconductor surface. The outer Helmholtz layer consists of solvent ions that accumulate to counterbalance the inner Helmholtz layer. Together, these layers form the Helmholtz layer.^34^ In contrast to the space-charge capacitance, the Helmholtz capacitance remains constant under varying applied potential.^28^ The Helmholtz capacitance typically has values around 20 × 10^–6^ F cm^–2^.^31^

*C*_sc_ and *C*_H_ are combined in a single capacitance *C*_cmb_,^31,32^ following Kirchhoff’s law

31 $$\frac{1}{C_{cmb}}=\frac{1}{C_{sc}\left(u^{eq}\right)}+\frac{1}{C_{H}}$$

In this context, the term *C*_bulk_ is sometimes used in the literature to refer to either *C*_sc_ or *C*_cmb_.^32,35^ To avoid confusion, the term *C*_bulk_ is not used here.

The capacitance *C*_cmb_ describes the ideal capacitive behavior. However, experimental EIS measurements are typically nonideal. To account for the nonidealities, the capacitance is replaced by a constant phase element (CPE). The impedance of the CPE is defined as follows

32 $$Z_{cmb,CPE}=\frac{1}{{\left(i\omega \right)}^{\alpha }Q_{cmb}}$$

where α [−] is the exponent and *Q*_cmb_(*u*_ρ_^eq^) [F cm^–2^ s^–(1−α)^] is the CPE prefactor, which represents a pseudocapacitance.

Following the equivalent circuit with CPE in Figure 1, the relationship between the impedances *Z*_cell_ and *Z*_SEI_ is described as follows

33 $$Z_{cell}\left(i\omega ,u^{eq}\right)=R_{s}+\frac{Z_{SEI}\left(i\omega ,u^{eq}\right)}{{\left(i\omega \right)}^{\alpha }Q_{cmb}\left(u^{eq}\right)Z_{SEI}\left(i\omega ,u^{eq}\right)+1}$$

### 3.3 Maximum Likelihood Estimator (MLE)

The estimation procedure aims to minimize the error between the modeled impedance *Z*_cell_(ω_*f*_,*u*_ρ_^eq^,**k**) and the experimentally measured impedance ${Ẑ}_{cell}\left(\omega_{f},u_{\rho }^{eq}\right)$ by adjusting the rate constants of the modeled impedance. This error is given by

34 $$ê\left(\omega_{f},u_{\rho }^{eq},k\right)=Z_{cell}\left(\omega_{f},u_{\rho }^{eq},k\right)-{Ẑ}_{cell}\left(\omega_{f},u_{\rho }^{eq}\right)$$

The impedance is measured *M* times (*M* independent realizations) at *F* different frequency pulsations ω_*f*_ with indices *f* ∈ {1, 2, ..., *F*}, and *P* steady-state potentials *u*_ρ_^eq^ with ρ ∈ {1, 2, ..., *P*}. The error equation includes the mean of the *M* impedance measurements: ${Ẑ}_{cell}\left(\omega_{f},u_{\rho }^{eq}\right)=\frac{1}{M}\sum_{m=1}^{M}Z_{cell}^{\left[m\right]}\left(\omega_{f},u_{\rho }^{eq}\right)$ . The errors at each frequency and potential are combined into a cost value, *V*(**k**, *Z*), which is typically determined as the sum of the squared error values. This cost value is minimized iteratively, which leads to an estimator of the rate constants k̂(Ẑ)∈

$$\mathrm{k̂}\left({Ẑ}_{cell}\right)=\underset{k}{\arg\min}V\left(k,{Ẑ}_{cell}\right)$$ 35

In this work, the maximum likelihood cost function is used, which weighs the error by the measurement uncertainty. Consequently, higher-quality measurements, with a higher signal-to-noise ratio (SNR), contribute more to the cost value, while lower-quality measurements are rejected. Note that quality depends not only on the SNR but also on which frequencies contribute the most to the parameter estimates.^36^ The maximum likelihood cost function is given as follows^37^

36 $$V\left(k,{Ẑ}_{cell}\right)=\sum_{\rho =1}^{P}\sum_{f=1}^{F}\frac{{\left|ê\left(\omega_{f},u_{\rho }^{eq},k\right)\right|}^{2}}{{\mathrm{\sigma ̂}}_{ê}^{2}\left(\omega_{f},u_{\rho }^{eq}\right)}$$

where the least squared error of eq 34 is weighed by the sample variance of the *M* impedance measurements

37 $${\mathrm{\sigma ̂}}_{ê}^{2}\left(\omega_{f},u_{\rho }^{eq}\right)=\frac{1}{M}\frac{1}{M-1}\sum_{m=1}^{M}{\left|Z_{cell}^{\left[m\right]}\left(\omega_{f},u_{\rho }^{eq}\right)-{Ẑ}_{cell}\left(\omega_{f},u_{\rho }^{eq}\right)\right|}^{2}$$

Note that the impedance measurements in this work are taken at different potentials in addition to the frequency measurements. The companion study to this work has pointed out that data from at least two potentials should be combined for the rate constants to be uniquely identifiable.^27^ Hence, in addition to frequency, the weighed error is summed over potentials in the maximum likelihood cost function, as shown in eq 36.

### 3.4 Minimization Procedure

The minimization problem in this work is nonconvex, i.e., it has local and global minima. To find the global minimum, minimization in this work follows a two-step procedure. In the first step, an initial minimization finds a “coarse” initial estimate of the rate constants. A variety of existing optimization algorithms are available to find the global minimum of nonconvex problems, such as the particle swarm algorithm,^38^ the genetic algorithm,^26^ or the evolutionary strategy algorithm (ES). The latter was shown to be the most effective for the model in this work^27^ and is therefore used to obtain the initial estimate in this work. The ES algorithm has been implemented in MATLAB, as detailed in the companion study, with the settings given in the Supporting Information.^27^

In the second step, a “fine“ estimate of the rate constants is obtained using the Levenbergh-Marquardt algorithm, for which the Jacobian matrix is calculated. This algorithm readily finds local minima and is therefore not suitable for performing initial coarse minimization. The following scaling measures are applied to the estimation: First, the frequencies are scaled by the median of their values to improve the numerical stability of the estimation;^37^ Second, the Jacobian matrix is scaled by the 2-norm of its columns to improve its conditioning;^37^ Third, during the second step, the rate constants are normalized by dividing by the 2-norm of their values from the initial optimization step.

### 3.5 Impedance Data

The workflow for obtaining experimental and synthetic impedance data, estimating the rate constants, and reconstructing the impedance from the estimated rate constants is shown in Figure 2. From the impedance data, experimental (*1a*) or synthetic (*1b*), the rate constants **k̂** are estimated following the two-step procedure detailed in Section 3.4 (2). Subsequently, the estimated rate constants are inserted into the OER model to reconstruct the impedance (3).

**Figure 2:**
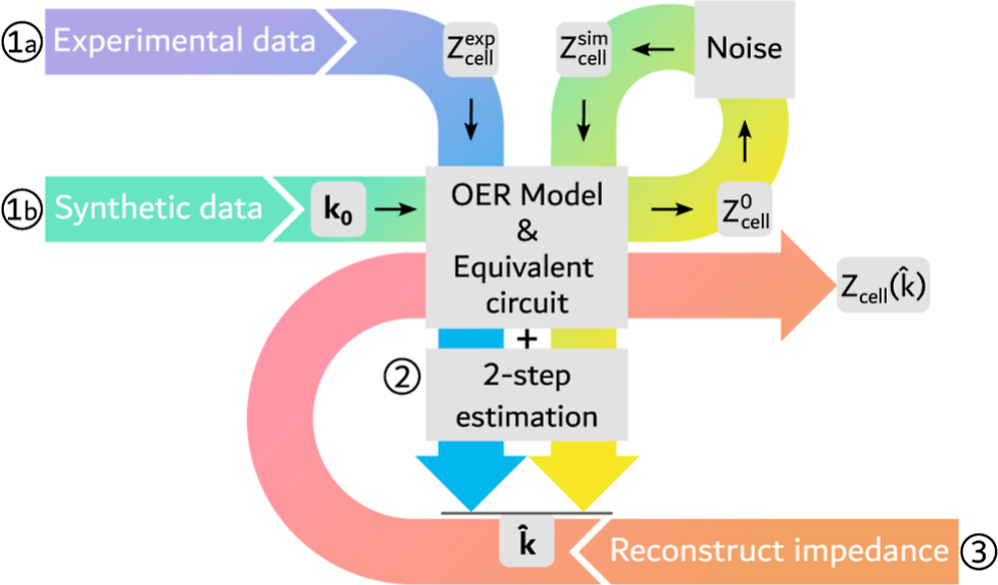
Estimation workflow centralized around the OER model and consisting of three steps: (1a) experimental data is obtained from EIS measurements or (1b) synthetic data is simulated using the model from predefined rate constants **k**_**0**_ and is then disturbed by noise; (2) the two-step estimation compares the input impedance data with the OER model to estimate the rate constants **k̂**; and (3) estimated rate constants are used as input to reconstruct the impedance $Z_{cell}\left(\mathrm{k̂}\right)$ .

#### 3.5.1 Synthetic Impedance Data

The synthetic impedance data is generated using the OER model in Section 3.1 and the equivalent circuit in Section 3.2 based on predefined rate constants **k**_0_ (*1b* in Figure 2). These constants are obtained from the theoretical expressions eqs 9 and 10, for which the Gibbs free energies are derived from DFT calculations for a hematite (Fe_2_O_3_) anode.^5^ The predefined rate constants are inserted into the microkinetic model and equivalent circuit to generate noiseless impedance measurements *Z*_cell,0_(ω_*f*_,*u*_ρ_^eq^), generated using a multisine potential excitation with *F* = 87 logarithmically distributed frequency components between 100 mHz and 100 kHz, for *P* = 4 potentials between 1.5 and 1.8 V, and M = 10 measurements generated at each frequency and potential. Subsequently, these are disturbed by noise with an SNR of 40 dB, providing the noisy synthetic measurements *Z*_cell_^[*m*]^(ω_*f*_,*u*_ρ_^eq^). All model parameters are listed in Supporting Information.

#### 3.5.2 Experimental Impedance Data

The experimental impedance data *Z*_cell_^exp^(ω_*f*_,*u*_ρ_^eq^) is obtained from EIS measurements on a three-electrode cell with an Fe_2_O_3_ anode (*1a* in Figure 2). FTO glass plates (FTO15, Pilkington) of 1.5 cm × 1.5 cm × 0.1 cm were used as substrate. They were cleaned sequentially in an ultrasonic bath (Branson 200) in 1:5 soap solution, acetone, and ethanol for 10 min each two times, followed by sonication in 1 M KOH for 10 min and sonication in DI water for 20 min, according to the procedure in.^39^ The clean FTOs were stored in a desiccator prior to deposition. Fe thin films with a thickness of 20 nm were deposited (XUV Optics laboratory, University of Twente, The Netherlands) by DC magnetron sputtering (Leybold Optics ADC A1105 UHV) (Fe target, base pressure of 1.2 × 10^–3^ mbar, 34.5 sccm Ar gas flow, operating power of 470 W). The Fe thin films were annealed under an ambient atmosphere at 645 °C for 10 min, with a ramping rate of 3 °C min^–1^ to oxidize to Fe_2_O_3_.

All EIS measurements were carried out in a Zahner photoelectrochemical cell (Zahner-Elektrik GmbH & Co. KG) using a BioLogic SP-150 potentiostat. An aqueous solution of 1 M NaOH (Supelco) was used as an electrolyte. A looped Pt wire and mini-HydroFlex hydrogen electrode (Gaskatel GmbH) were used as the counter and reference electrodes, respectively. Illumination was performed using an AM 1.5G solar simulator (Oriel LCS-100) operated with a 100 W Xe lamp with a calibrated illumination intensity of 100 mW cm^–2^ at the sample position (Newport 91150V reference cell). The experimental EIS measurements were carried out on *F* = 68 frequencies between 100 mHz and 300 kHz and on *P* = 4 potentials between 1.5 and 1.8 V vs RHE, with M = 5 measurements generated at each frequency and potential (for variance estimation).

#### 3.5.3 Limitations of the Experimental Data

The experimental impedance, *Z*_cell_^[*m*]^(ω_*f*_,*u*_ρ_^eq^), is calculated as the ratio between the applied potential signal, *U*_cell_(ω_*f*_,*u*_ρ_^eq^), and the current density signal, *J*_cell_(ω_*f*_,*u*_ρ_^eq^). Both signals carry measurement uncertainty, which is assumed to be zero-mean circular complex normally distributed (CCND):^37^ $\left[N_{U}\left(\omega_{f},u_{\rho }^{eq}\right),N_{J}\left(\omega_{f},u_{\rho }^{eq}\right)\right]∼N\left(0,\sigma^{2}\right)$ . The EIS software of the Biologic SP-150 potentiostat used in this work calculates the ratios internally, presenting to the user only the impedance *Z*_cell_^[*m*]^(ω_*f*_,*u*_ρ_^eq^), for which the uncertainty is noncircular. This section shows, through an example using synthetic impedance data, that the availability of only the impedance measurements has an adverse effect on the accuracy of the estimation, such as for the rate constants described in this paper.

In the cost function (eq 36), the mean of *M* impedance measurements, ${Ẑ}_{cell}\left(\omega_{f},u_{\rho }^{eq}\right)$ , is used. The mean can be calculated from applied potential and current density signals in different ways, of which two are given here. First, in the “simple approach” in eq 38, the mean of *M* measurements is computed after division of the potential and current signals. Second, in the “errors-in-variables” approach in eq 39, the mean of potential and current signals is computed before division.^37^

38 $${Ẑ}_{sa}\left(\omega_{f},u_{\rho }^{eq},M\right)=\frac{1}{M}\sum_{m=1}^{M}\frac{U^{\left[m\right]}\left(\omega_{f},u_{\rho }^{eq}\right)}{J^{\left[m\right]}\left(\omega_{f},u_{\rho }^{eq}\right)}$$

39 $$\begin{array}{l} {Ẑ}_{ev}\left(\omega_{f},u_{\rho }^{eq},M\right)=\frac{Û\left(\omega_{f},u_{\rho }^{eq},M\right)}{Ĵ\left(\omega_{f},u_{\rho }^{eq}\right)}\\ =\frac{\frac{1}{M}\sum_{m=1}^{M}U^{\left[m\right]}\left(\omega_{f},u_{\rho }^{eq},M\right)}{\frac{1}{M}\sum_{m=1}^{M}J^{\left[m\right]}\left(\omega_{f},u_{\rho }^{eq},M\right)} \end{array}$$

The errors-in-variables approach is typically used in maximum likelihood estimation, as it is essential that the uncertainty of ${Ẑ}_{ev}$ is zero-mean CCND. However, in practice, only the simple approach, ${Ẑ}_{sa}\left(\omega_{f},u_{\rho }^{eq},M\right)$ , can be used because the EIS potentiostat software typically computes $\frac{U^{\left[m\right]}\left(\omega_{f},u_{\rho }^{eq}\right)}{J^{\left[m\right]}\left(\omega_{f},u_{\rho }^{eq}\right)}$ internally. The uncertainty of this fraction is not zero-mean CCND, which is detailed in Supporting Information. Consequently, this causes a systematic error in the impedance estimator. Although alternative measurement methods, such as current–voltage measurements, can provide both the potential and the current density, these methods are typically not designed to accept sinusoidal excitation signals.

To visualize this systematic error, an example is provided using synthetic impedance data showing the convergence of the estimators as the number of measurements *M* increases. Relative estimators are determined by dividing the estimators by the synthetic impedance prior to the addition of noise $Z_{0}\left(\omega_{fc},u_{\rho c}^{eq}\right)=\frac{U_{0}\left(\omega_{fc},u_{\rho c}^{eq}\right)}{J_{0}\left(\omega_{fc},u_{\rho c}^{eq}\right)}$ . In Figure 3, the relative estimators ${Ẑ}_{sa}\left(\omega_{fc},u_{\rho c}^{eq},M\right)$ (simple approach) and ${Ẑ}_{ev}\left(\omega_{fc},u_{\rho c}^{eq},M\right)$ (errors-in-variables approach) are shown for *M* ∈ [10^1^, 10^5^]. For this example case, ω_fc_ = 0.6 rad s^–1^ and *u*_ρc_^eq^ = 1.6 V.

**Figure 3:**
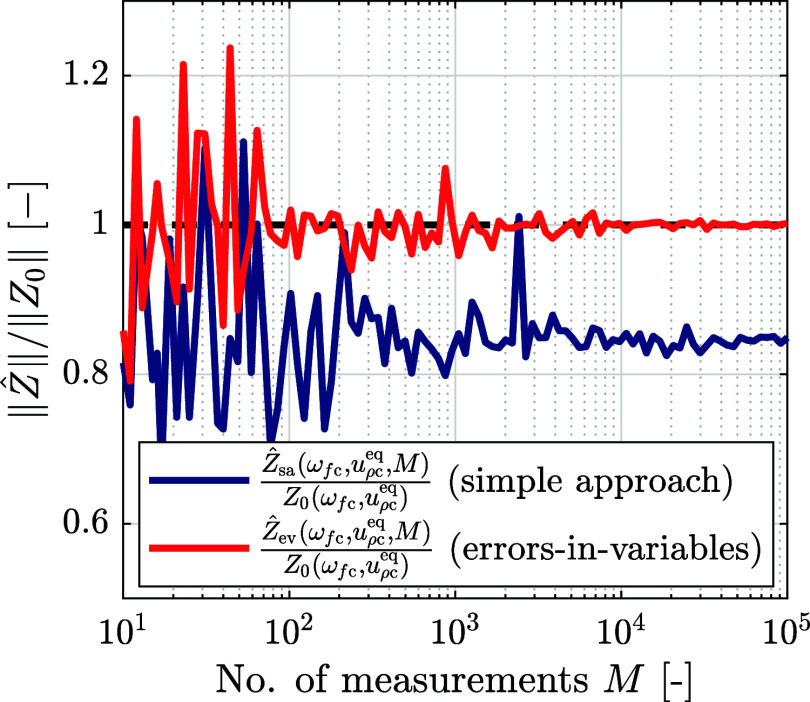
Relative estimators of the impedance ${Ẑ}_{sa}\left(\omega_{fc},u_{\rho c}^{eq},M\right)$ and ${Ẑ}_{ev}\left(\omega_{fc},u_{\rho c}^{eq},M\right)$ divided by the noiseless impedance, *Z*_0_(ω_fc_,*u*_ρc_^eq^), as a function of the number of measurements, *M* ∈ [10^1^, 10^5^].

Figure 3 shows that both relative estimators converge as *M* increases; however, the values to which the two approaches converge are different. While the relative estimator of ${Ẑ}_{ev}\left(\omega_{fc},u_{\rho c}^{eq},M\right)$ converges to 1 and thus coincides with the noiseless (correct) impedance value, the relative estimator of ${Ẑ}_{sa}\left(\omega_{fc},u_{\rho c}^{eq},M\right)$ converges to a value ≠ 1, exhibiting a systematic error with respect to the noiseless impedance, *Z*_0_(ω_*f*c_,*u*_ρc_^eq^). The source of this error is further detailed in the Supporting Information, where it is also shown that the size of the error depends on the signal-to-noise ratio (SNR) of the measurements.

## 4 Results

This section presents the rate constant estimation results. First, the results obtained from synthetic measurement data are presented. Second, the uncertainty of the synthetic estimation was analyzed across various potentials and frequencies. Third, the absence of model errors was assessed using a validation test based on the cost function. Finally, the results of the estimation using experimental EIS data are presented.

### 4.1 Estimation Using Synthetic Data

The two-step estimation procedure is applied to synthetic EIS data generated with predefined rate constants **k**_0_ (step 2 in Figure 2). These rate constants are shown together with the estimated rate constants **k̂** in Table 1. The percentage relative error is calculated as follows

40 $$\epsilon_{k}=\frac{\left|k_{0}-\mathrm{k̂}\right|}{k_{0}}\times 100\%$$

**Table 1:** Predefined Rate Constants **k**_0_, Estimated Rate Constants **k̂**, and the Percentage Relative Error ϵ_k,1_

	**k**_0_	${\mathrm{k̂}}^{sim}$	ϵ_k,1_ [%]
*k*_f1_	4.69 × 10^–17^	4.89 × 10^–17^	4.27 × 10^0^
*k*_b1_	9.80 × 10^–28^	9.77 × 10^–28^	3.29 × 10^–1^
*k*_f2_	4.40 × 10^–20^	4.40 × 10^–20^	7.36 × 10^–3^
*k*_b2_	2.19 × 10^–21^	2.19 × 10^–21^	2.48 × 10^–2^
*k*_f3_	1.56 × 10^–16^	1.56 × 10^–16^	3.88 × 10^–2^
*k*_b3_	1.48 × 10^–31^	–5.090 × 10^–29^	3.46 × 10^4^
*k*_f4_	1.47 × 10^–19^	1.47 × 10^–19^	4.96 × 10^–3^

The errors between the estimated and predefined rate constants are small (below 1%), with the exception of *k*_f1_ (below 5%) and *k*_b3_, which has a significant error of 3.46 × 10^4^%.

To investigate the robustness of the results in Table 1, the estimation is repeated a total of 30 times (*N*_est_ = 30), each with different realizations of the random noise applied to the synthetic impedance data. Three of these estimations did not converge, as they were limited by the maximum number of iterations and are excluded from the following results. Figure 4 illustrates the percentage relative error ϵ_k,*n*_ (blue circles) for *n* ∈ {1, ..., *N*_est_}, as well as the average percentage relative error ${\mathrm{\epsilon ̅}}_{k}$ of these estimates (red ×). For each rate constant, the relative errors are generally distributed within a range of 2 orders of magnitude. For most rate constants, the average relative error is generally low, around the order of 1 or less. A notable exception is the large average relative error of ${\mathrm{k̂}}_{b3}$ , around ${\mathrm{\epsilon ̅}}_{k_{b3}}=$ 4.77 × 10^5^ %. This result is further discussed in this section.

**Figure 4:**
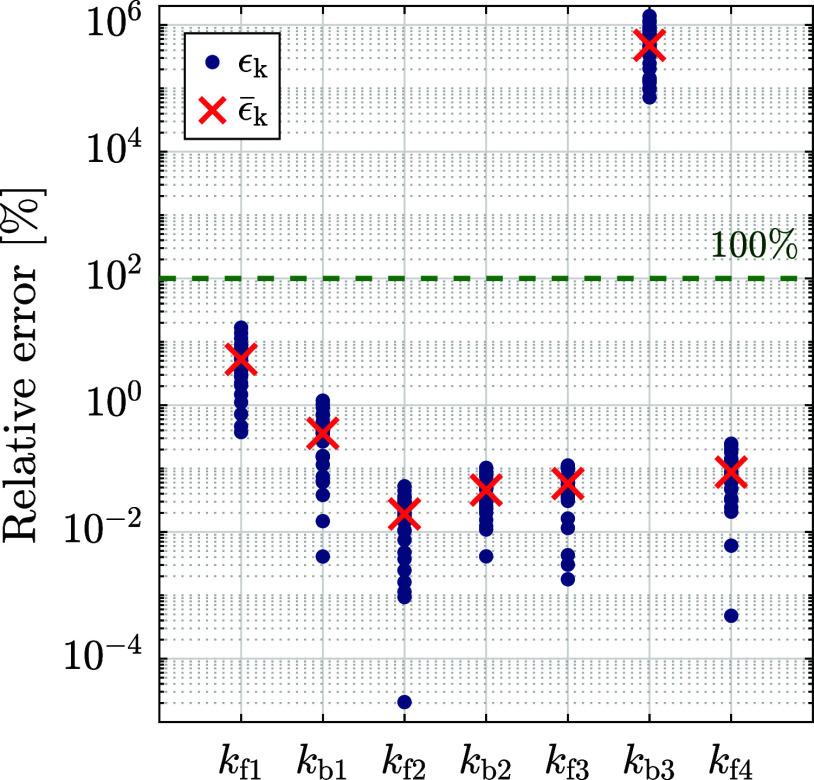
Percentage relative error ϵ_k,*n*_ (blue circle) between the estimated rate constants ${\mathrm{k̂}}_{n}$ and predefined rate constants **k**_0_ for *n* ∈ {1, ..., *N*_est_} with *N*_est_ = 30. The average percentage relative error for each rate constant is shown as ${\mathrm{\epsilon ̅}}_{k}$ (red ×). 100% relative error is indicated as a dashed line.

Additionally, we investigate the quality of the estimation by reconstructing the surface coverages of the intermediate species using the estimated rate constants $\theta_{i}\left({\mathrm{k̂}}_{n}\right)$ according to (eqs 25–28) and comparing these to the coverages calculated with the predefined rate constants θ_*i*_(**k**_0_). Figure 5 shows these reconstructed coverages $\theta_{i}\left({\mathrm{k̂}}_{n}\right)$ (markers), and the coverages calculated from the predefined rate constants θ_*i*_(**k**_0_) (dashed lines) as a function of the applied potential for 30 estimations. The reconstructed coverages $\theta_{i}\left({\mathrm{k̂}}_{n}\right)$ are well-aligned with the predefined coverages, with a maximum error of 9.63 × 10^–4^. This error is very small, and this result proves that the coverages can be estimated well from synthetic EIS data using our methodology.

**Figure 5:**
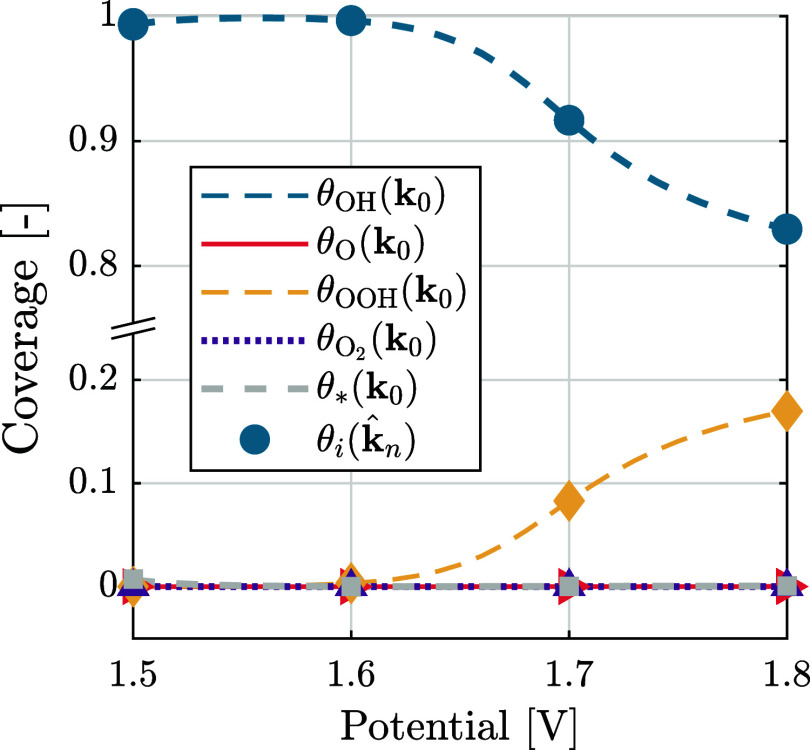
Species coverages θ_*i*_(**k**_0_) and coverage of empty surface sites θ_*_(**k**_0_) calculated from the predefined rate constants **k**_0_ (lines) and coverages $\theta_{i}\left({\mathrm{k̂}}_{m}\right)$ calculated from the estimated rate constants ${\mathrm{k̂}}_{n}$ with *n* ∈ {1, ..., *N*_est_} for *N*_est_ = 30 repeated estimations (markers).

### 4.2 Analysis of Model and Estimation Uncertainty

In order to generalize the observations made from the estimation, the Cramer-Rao lower bound (CRLB) is determined.^37^ The CRLB represents the theoretical minimum achievable variance for the rate constants and can be compared to the variance of the rate constants obtained using the MLE. A higher CRLB corresponds to a larger variance and hence to a potentially less precise estimation. Since the MLE is asymptotically optimal with an increasing number of measurements, the variance of the MLE approaches the CRLB as the number of impedance measurements at each frequency and potential, *M*, approaches infinity.

In the following analysis of the CRLB, the pseudocapacitance of the constant phase element (CPE), **Q**_**cmb**_, and the CPE exponent, **α**, are included, as they can be estimated simultaneously with the rate constants. As stated previously, the CPE is associated with nonideal capacitive behavior. The combination of estimation parameters (rate constants, CPE pseudocapacitances, and CPE exponents) is termed **γ** ∈ [**k**, **Q**, **α**]. The results of a simultaneous estimation of the rate constants, the pseudocapacitances of CPE, and the exponents of CPE are given in Supporting Information.

The standard deviation of each estimation parameter is determined over *M* = 10 synthetic measurements, $\sigma_{\gamma }\left(\mathrm{\gamma ̂},\Theta \right)$ , with Θ­(ω_*f*_,*u*_ρ_^eq^)=(*U*(ω_*f*_,*u*_ρ_^eq^),*Y*(ω_*f*_,*u*_ρ_^eq^)). The standard deviation is divided by the predefined value of the respective estimation parameter to give the relative standard deviation (RSD). This RSD is shown against the potential in Figure 6 (solid lines). Furthermore, the CRLB *C*_r_(γ_0_, Θ) is determined for each estimation parameter, and its square root is similarly divided by the respective predefined estimation parameter (dashed gray lines in Figure 6). Note that the RSD shown in Figure 6 is calculated individually for each potential, while in the estimation in Section 4.1 all potentials are combined. This results in a single RSD per rate constant that is lower than for the separate potentials (lowest for $\sigma_{\gamma }\left({\mathrm{k̂}}_{f2},\Theta \right)/k_{f2}=1.12\times 10^{-4}$ and highest for $\sigma_{\gamma }\left({\mathrm{k̂}}_{b3},\Theta \right)/k_{b3}=1.64\times 10^{3}$ ).

**Figure 6:**
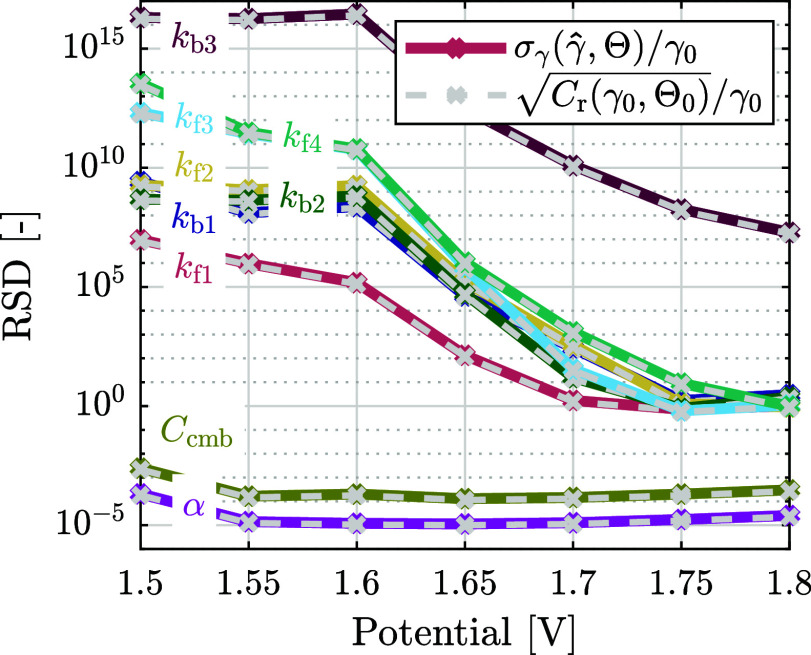
Relative standard deviation (RSD) of each estimation parameter $\sigma_{\gamma }\left(\mathrm{\gamma ̂},\Theta \right)/\gamma_{0}$ is shown over potential (solid lines). Additionally, the square root of the CRLB $\sqrt{C_{r}\left(\gamma_{0},\Theta \right)}$ for each rate constant divided by the respective predefined rate constant in **γ**_0_ is shown over potential (dashed gray lines). The RSD is obtained by taking the standard deviation of the estimation $\sigma_{\gamma }\left(\mathrm{\gamma ̂},\Theta \right)$ for each estimation parameter **γ** ∈ [**k**, **Q**, **α**] (rate constants **k**, CPE pseudocapacitances **Q**, and CPE exponents **α**) and dividing by the predefined value of the respective estimation parameter in **γ**_0_.

Four observations are given in Figure 6. First, the RSD of the estimation (solid lines) is close to the relative square root of the CRLB (dashed lines). This indicates that the uncertainty from *M* = 10 measurements is close to the minimally achievable uncertainty, as represented by the CRLB.

Second, the RSD of all rate constants decreases with increasing potential. This could be explained by observing the change in species coverages with potential in Figure 5. Initially, with increasing potential, the surface becomes completely covered with θ_OH_, and with further increasing potential, the surface becomes partially covered with θ_OOH_. This suggests that at lower potentials only the dynamics of θ_OH_ are significant, which is given by the first two equations in the mass balance (12). With increasing potential, also the dynamics of θ_OOH_ becomes significant, related to the equation of θ̇_3_ in eq 12. This aligns with Vandeputte et al. (2025), where it is observed that a reduced model of only the first two equations in eq 12 is a sufficient approximation at lower potentials, while at higher potentials all three equations in eq 12 are necessary.^27^ A lower RSD corresponds to a smaller estimation uncertainty, making higher potentials favorable for estimation. Note furthermore that the rate constant *k*_f1_, related to the formation of θ_OH_ has the smallest RSD of the rate constants throughout the lower potentials, since only θ_OH_ is significant in this range.

Third, the relative square root of the CRLB of the rate constant *k*_b3_ is, according to Figure 6, significantly higher compared to the other rate constants. This corresponds to the relatively large estimation error for *k*_b3_ (see Figure 4 and Table 1) and indicates that it is difficult to accurately identify *k*_b3_ in the investigated range of potentials.

Finally, the RSD of the CPE coefficients (exponent **α** and pseudocapacitance **Q**) is notably lower than the RSD of the rate constants even at high potentials. At 1.9 V vs RHE, where the difference is minimal, the RSD of *k*_f3_ (the smallest rate constant at that potential) is ∼3 × 10^3^ times larger than that of *C*_cmb_. At 1.6 V vs RHE, the RSD ratio of *k*_f1_ and *C*_cmb_ is ∼4 × 10^9^. This difference indicates that these CPE coefficients are more readily identifiable than the rate constants throughout the potential range. Conversely, variations of the CPE coefficients will have a stronger effect on the impedance than the rate constants.

The CRLB can also be used to analyze the influence of frequency on the sensitivity of the estimation parameters. Figure 7 shows the relative square root of the CRLB of each estimation parameter, determined at 1.8 V. The CRLB is computed using impedance data at 9 subsequent frequencies. Each CRLB data point in Figure 7 is placed at the median of the 9 frequencies.

**Figure 7:**
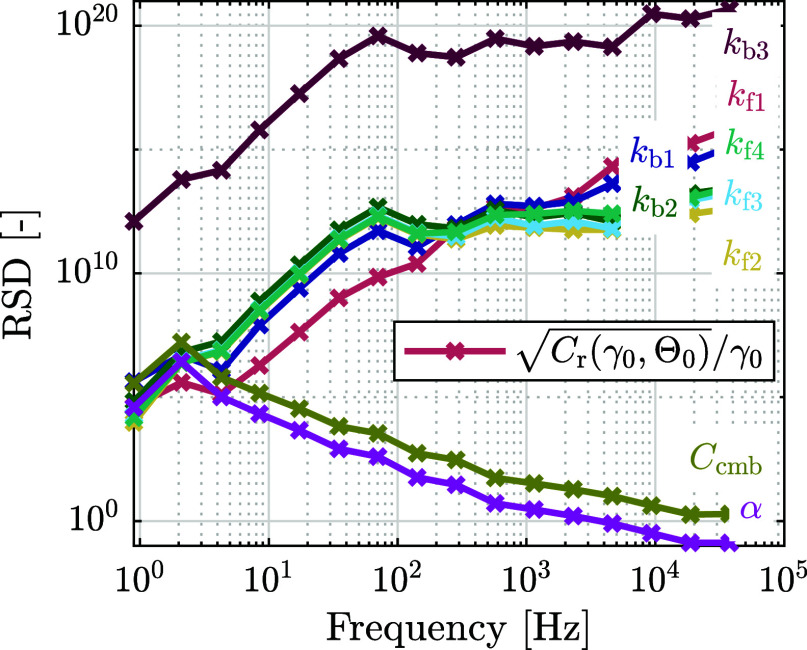
Relative square root of the CRLB $\sqrt{C_{r}\left(\gamma_{0},\Theta \right)}/\gamma_{0}$ for each rate constant as a function of frequency. The relative CRLB is determined at 1.8 V. At each frequency in the figure, impedance data at 9 subsequent frequencies are used. The CRLB data point is placed at the median of these 9 frequencies.

Figure 7 shows an inverse effect of frequency on the relative CRLB of the rate constants and CPE coefficients: For the rate constants, the relative CRLB increases with increasing frequency, whereas for the CPE coefficients, the relative CRLB decreases with increasing frequency. This can be related to the parallel branches in the equivalent circuit, which split the total current over the cell. At low frequency, the impedance of the CPE is high, such that *Z*_cmb,CPE_ ≫ *Z*_SEI_, causing the current to mainly pass through *Z*_SEI_. As a result, the impedance of the cell is more sensitive to variations in the rate constants. With an increase in frequency, the current increasingly passes through *Z*_cmb,CPE_. Thus, at higher frequency, the model is more sensitive to the CPE coefficients and less to the rate constants. Note that the CRLB values in Figure 7 do not correspond to the values at 1.8 V in Figure 6, as the values in the latter figure are derived from the full frequency range, unlike for the values in Figure 7, which are derived from only 9 frequencies.

The effect of the frequency range on the identifiability of the rate constants was further examined using the cost value (eq 36) by varying the rate constants slightly above or below the predefined values **k**_**0**_ and calculating the corresponding cost values. Logically, the cost value should be at a minimum when it is equal to the value of **k**_**0**_. However, if the variation in the cost value is negligible, it becomes difficult to identify the minimum. Here, the rate constants *k*_b3_ and *k*_f1_ are compared. Figure 8 shows the cost value when *k*_b3_ (a) and *k*_f1_ (b) vary around their predefined values, *k*_b3,0_ and *k*_f1,0_, respectively. Furthermore, this is shown as a function of the minimum of the frequency range of the EIS measurements, as the corresponding dynamics could lie outside this range. For *k*_b3_, the cost value remains approximately constant with changes in the value of *k*_b3_, i.e., there is no change in color in the vertical direction in Figure 8, when the minimum frequency exceeds 1 × 10^–4^ Hz. A variation in the cost with changes in the value of *k*_b3_ can only be discerned for a minimum frequency around 1 × 10^–5^ Hz (at this frequency, there is a color gradient in the vertical direction in Figure 8). This suggests that its dynamics are low frequency, in the order of 1 × 10^–5^ Hz, which is well below the typical experimental EIS measurement frequencies and the physical limitations of the potentiostat. We have taken 1 × 10^–1^ Hz as a measurement limit (indicated with a dashed line in Figure 8). We can therefore conclude that rate constant *k*_b3_, given its predefined value, cannot be accurately identified in the measurable frequency range. In contrast, for varying *k*_f1_, the cost value has a clear minimum at the predefined value *k*_f1,0_, even within the measurable frequency range. Hence, *k*_f1_ is identifiable in the measurable frequency range.

**Figure 8:**
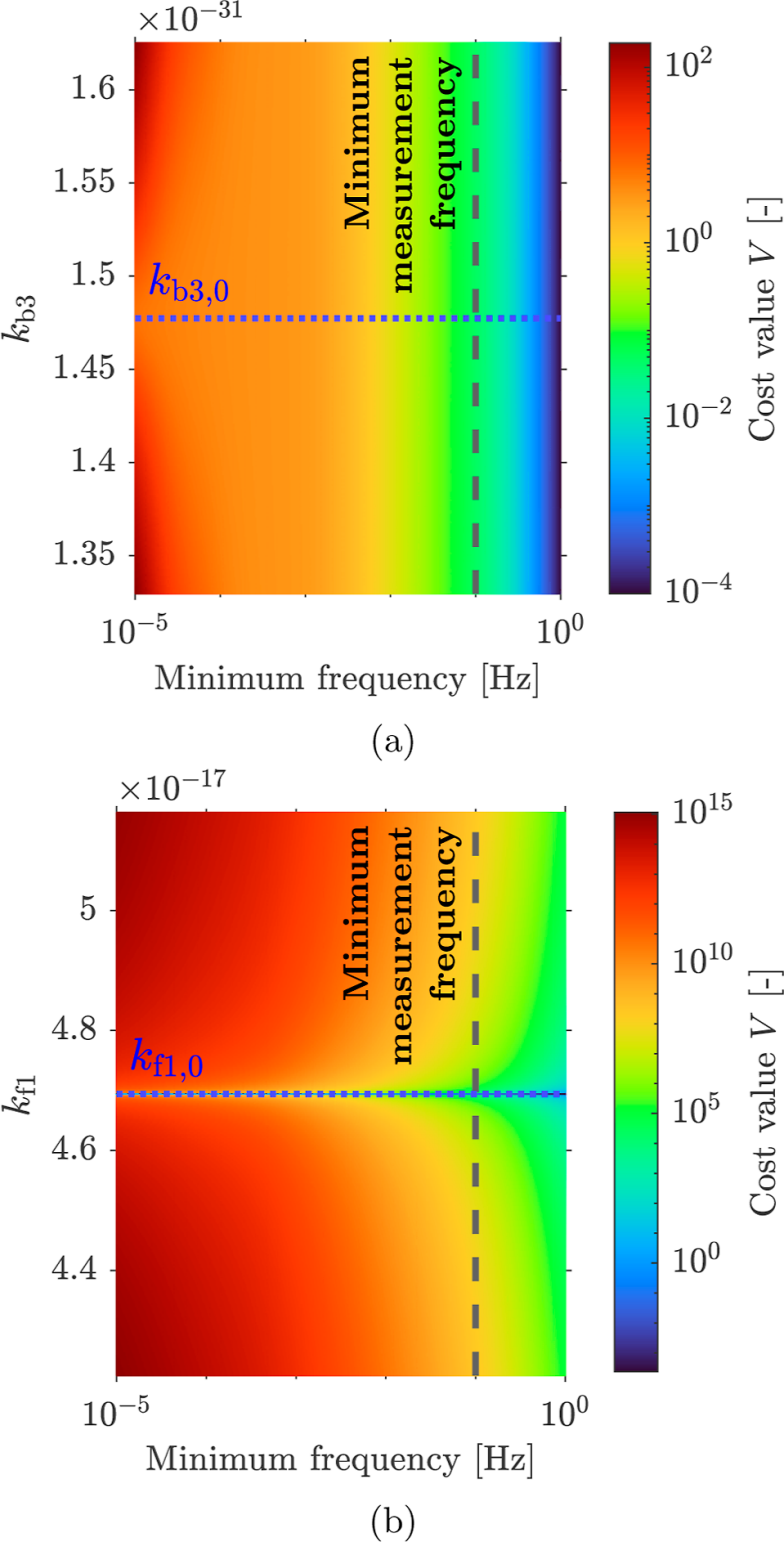
Cost value *V* calculated as a function of (a) *k*_b3_ and (b) *k*_f1_ near the predefined values *k*_b3,0_ and *k*_f1,0_, and as a function of the minimum frequency of the EIS data. The lowest measured frequency of the experimental measurements is indicated (dashed line). At this frequency, (a) no gradient in the cost value can be discerned, and (b) cost values are at a minimum at the predefined *k*_f1,0_.

### 4.3 Model Validation Test

A cost function model validation test is used to detect model errors.^37^ The theoretical minimum value of the maximum likelihood cost function *V*_min_ is related to the number of frequencies *F* and the number of free estimation parameters *n*_*k*_ divided by 2, as each frequency has two known components, the amplitude and phase, or equivalently, the real and imaginary part.^37^ The cost function (36) in this work is additionally summed over multiple potentials *P*. Therefore, the number of potentials is novelly incorporated in the model validation test as follows

41 $$V_{\min}=F\cdot P-\frac{n_{k}}{2}$$

Considering that each estimate is derived from a limited number of *M* measurements, a correction must be made to include the resulting uncertainty, which gives the expected value $E\left\{V_{\min}\right\}$ and variance var­{*V*_min_}. These are then used to create the confidence bound of the cost function $C_{bnd}\left(P\right)$ with confidence P^37^

42 $$E\left\{V_{\min}\right\}=\frac{M-1}{M-2}V_{\min}$$

43 $$var\left\{V_{\min}\right\}=\frac{{\left(M-1\right)}^{3}}{\left(M-3\right){\left(M-2\right)}^{2}}V_{\min}$$

44 $$C_{bnd}\left(P\right)=E\left\{V\right\}\pm \sqrt{2var\left\{V\right\}}{\mathrm{erf}}^{-1}\left(P\right)$$

If the MLE cost value (eq 36) at the global minimum is significantly higher than the confidence bound, this could indicate model errors. On the other hand, a cost value lower than the confidence bound could indicate incorrect modeling of the measurement uncertainty.

The confidence bound is used to validate the OER model using the repeated estimations, *N*_est_ = 30, from Section 4.1. Figure 9 shows for *F* = 87, *P* = 4, M = 10, and *n*_*k*_ = 7 the expected value of the cost function $E\left\{V\right\}=3.88\times 10^{2}$ (black dot), the confidence bound $C_{bnd}\left(P\right)=\left[3.41\times 10^{2},4.34\times 10^{2}\right]$ with confidence P=0.95 (black dashed lines), and the cost function values of the estimates $V\left({\mathrm{k̂}}_{n}\right)$ , *n* ∈ {1, ..., *N*_est_} (blue ×). The values of the cost function, $V\left({\mathrm{k̂}}_{n}\right)$ , are within the confidence bounds, $C_{bnd}\left(P\right)$ . Hence, the model is validated with respect to this test.

**Figure 9:**
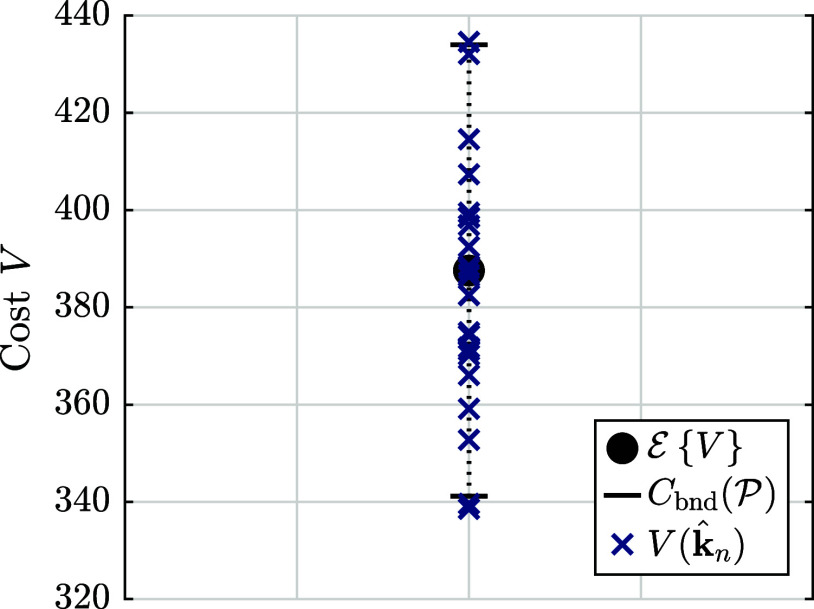
Expected value of the cost function E{V} (black dot), the confidence bound $C_{bnd}\left(P\right)$ with confidence P=0.95 (black dashed lines), and the cost function values $V\left({\mathrm{k̂}}_{n}\right)$ for the *n* ∈ {1, ..., *N*_est_} estimations from Section 4.1 (blue ×).

### 4.4 Estimation Using Experimental Data

This section investigates the rate constant estimation from experimental EIS measurements *Z*_cell_^exp^(ω_*f*_,*u*_ρ_^eq^) described in Section 3.5.2. All rate constants will be estimated, except for *k*_b3_, which was revealed in the previous section to be unidentifiable. The rate constants from the model, *Z*_SEI_(ω_*f*_,*u*_ρ_^eq^), are estimated from the experimental EIS measurement data, *Z*_cell_^exp^(ω_*f*_,*u*_ρ_^eq^) (step 2 in the estimation workflow in Figure 2). For the estimation, values need to be derived for the other components in the equivalent circuit in Figure 1 apart from *Z*_SEI_(ω_*f*_,*u*_ρ_^eq^). These values are derived as follows: The series resistance, *R*_s_, is extracted from the data directly as the value where the experimental EIS data crosses the *x*-axis at the highest frequency. The series resistance value is given in Supporting Information Figure S2. The CPE exponent is fixed at α = 0.89, which is based on the semicircle curvature in the Nyquist diagram. This indicates that the measured processes are more complex than exclusively diffusion (α = 0.5) or ideal capacitance (α = 1). The CPE pseudocapacitance is estimated for varying potentials $\mathrm{Q̂}\left(u_{\rho }^{eq}\right)$ . The rate constant estimation was performed five times with randomized starting values for the evolutionary strategy algorithm. Among these five instances, the estimate with the lowest cost value V(k̂,Q̂) is further analyzed. The estimated pseudocapacitances are given in Supporting Information Figure S2.

The rate constants of the estimation with the lowest cost value V(k̂,Q̂) are shown in Table 2 together with the predefined rate constants, **k**_0_ (obtained from DFT calculations). It is found that the forward rate constants are larger than the backward rate constants of each step. A quantitative comparison reveals that most estimated and predefined rate constants are approximately within two to 3 orders of magnitude. The exception is the rate constant *k*_b2_, which has an erroneously negative estimate. As stated in Section 1, differences in rate constants can follow from differences in interfacial conditions between the experimental measurements and the DFT calculations, which assume an ideal surface (hence no defects, pores, and impurities) and near-zero temperatures. Furthermore, the predefined rate constants are influenced not only by the DFT-derived free energies but also by additional parameters, as follows from eqs 9 and 10. In our earlier study, we demonstrated that the valence band energy level, *E*_V_, and the reorganization energy, λ, in particular affect the steady-state current density.^40^ As the current density is determined from the rate constants, see eqs 15–17, we expect these parameters to have a similar influence on the rate constants.

**Table 2:** Predefined Rate Constants **k**_0_ and Estimated Rate Constants ${\mathrm{k̂}}^{\exp}$ From Experimental Impedance *Z*_exp_

	**k**_0_	${\mathrm{k̂}}^{\exp}$
*k*_f1_	4.69 × 10^–17^	2.48 × 10^–18^
*k*_b1_	9.80 × 10^–28^	3.88 × 10^–26^
*k*_f2_	4.40 × 10^–20^	9.29 × 10^–23^
*k*_b2_	2.19 × 10^–21^	–1.23 × 10^–30^
*k*_f3_	1.56 × 10^–16^	2.22 × 10^–16^
*k*_f4_	1.47 × 10^–19^	7.88 × 10^–18^

From the estimated rate constants, ${\mathrm{k̂}}^{\exp}$ , in Table 2, the impedance $Z_{cell}\left({\mathrm{k̂}}^{\exp}\right)$ is reconstructed (step 3 in the estimation workflow in Figure 2). The reconstructed impedance data is presented alongside the experimental impedance data, *Z*_cell_^exp^(ω_*f*_,*u*_ρ_^eq^), in Figure 10. In the figure, the impedance data is represented as a Nyquist diagram showing a) the full spectrum and b) a zoom in on the higher potentials and as a Bode diagram showing c) the magnitude |*Z*| [Ω], and d) the phase ∠*Z* [°]. The experimental data, *Z*_cell_^exp^(ω_*f*_,*u*_ρ_^eq^), is shown as dots in the Nyquist diagrams (Figure 10a,b) and as straight lines in the Bode diagram (Figure 10c,d) for applied potentials between 1.5 and 1.8 V vs RHE. The reconstructed data, $Z_{cell}\left({\mathrm{k̂}}^{\exp}\right)$ , is visualized as a dashed line in all plots of Figure 10.

**Figure 10:**
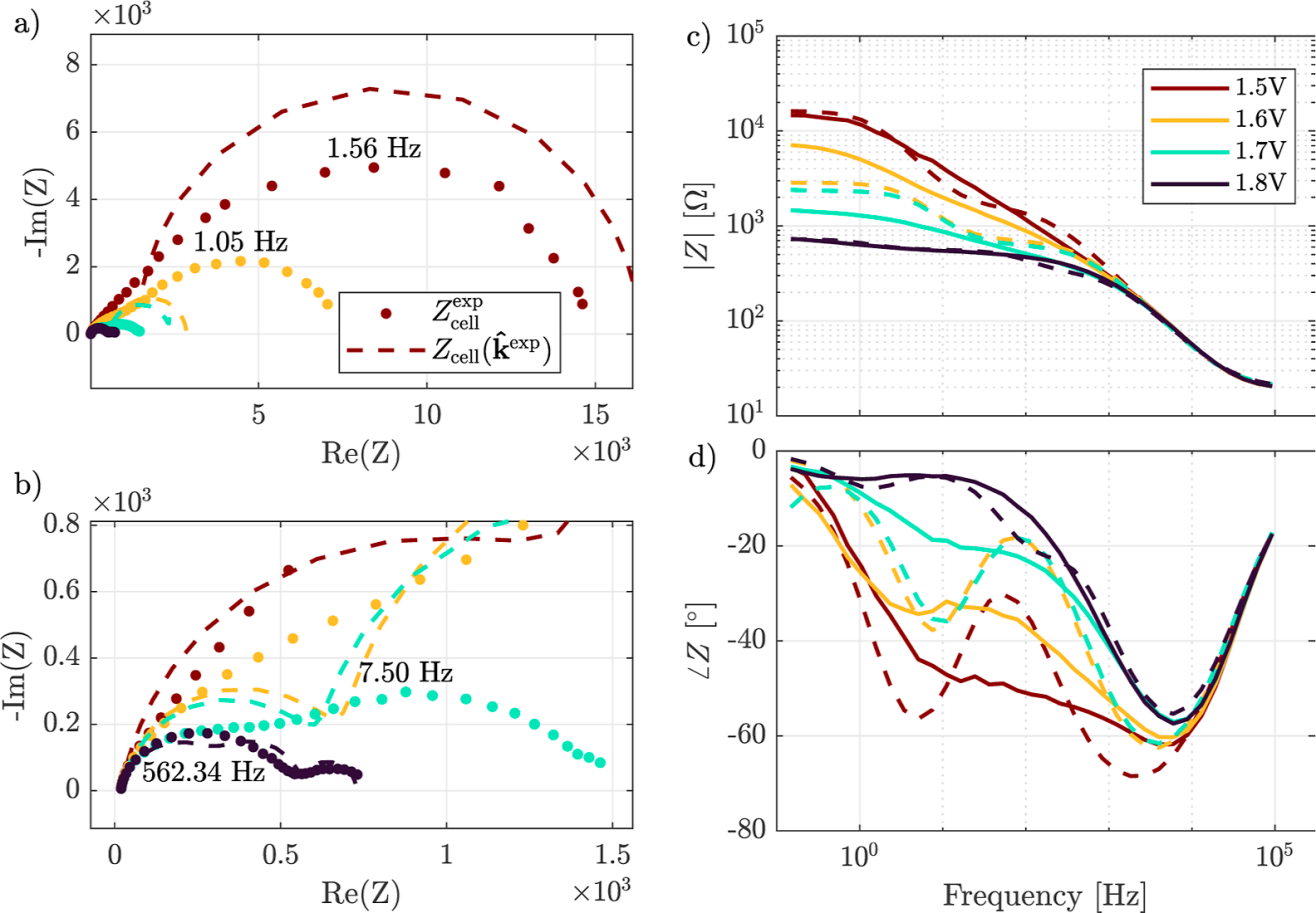
Experimental impedance measurements, with the mean taken over *M* measurements, *Z*_cell_^exp^, and the impedance reconstructed from the estimated rate constants, $Z_{cell}\left({\mathrm{k̂}}^{\exp}\right)$ . The data is represented as a Nyquist diagram showing (a) full spectrum and (b) zoom in on the higher potentials, and as a Bode diagram with (c) magnitude |*Z*| [Ω] and (d) phase ∠*Z* [°]. The experimental data, *Z*_cell_^exp^(ω_*f*_,*u*_ρ_^eq^), is shown in the Nyquist diagrams (a,b) as dots and in the Bode diagram (c,d) as solid lines for applied potentials between 1.5 and 1.8 V vs RHE. The reconstructed data, $Z_{cell}\left({\mathrm{k̂}}^{\exp}\right)$ , is shown in all plots as a dashed line.

The experimental data, in both the Nyquist and Bode representations, indicate that several processes are present. In the Nyquist diagram of the experimental data, these processes appear as partially overlapping, depressed semicircles. This data aligns well with similar measurements obtained in previous work.^10^ The reconstructed impedance shows these different processes even more pronounced: clearly separated, multiple semicircles are seen in the Nyquist diagrams (Figure 10a,b). This is expected because the electrochemical model, represented as the impedance *Z*_SEI_ in Figure 1, simulates four distinct OER reaction steps.

For all potentials, the experimental and reconstructed impedances align at higher frequencies, above 1 × 10^4^ Hz (see Figure 10c,d). As stated in Section 4.2, the higher frequency range is likely associated with the CPE, suggesting a reasonable estimate for the CPE pseudocapacitances.

At frequencies below 1 × 10^4^ Hz, a significant mismatch can be observed between the reconstructed and experimental impedances, although a relatively good alignment can be found between the impedances at 1.8 V vs RHE (see again Figure 10c,d). This mismatch is because the experimental impedances exhibit depressed semicircles, whereas the reconstructed impedances do not. A possible cause of these depressed semicircles might be diffusive transport processes,^41^ which are not taken into account in the model in this manuscript. However, ongoing efforts by the authors are focused on extending the model with a description of the dynamics of the semiconductor charge carriers. This will provide a thorough description of charge carrier diffusion, and more realistic values for the rate constants can be estimated.

From the estimated rate constants, ${\mathrm{k̂}}^{\exp}$ , the species coverages are also reconstructed $\theta \left({\mathrm{k̂}}^{\exp},u^{eq}\right)$ , according to eqs 25–29. These reconstructed coverages are given as a function of potential in Figure 11 and can be compared to the coverages derived from the predefined rate constants θ­(**k**_**0**_, *u* ^eq^) shown in Figure 5. The coverages reconstructed from the estimated rate constants and from the predefined rate constants show significantly different behavior: Although for both $\theta \left({\mathrm{k̂}}^{\exp},u^{eq}\right)$ and θ­(**k**_**0**_, *u* ^eq^), θ_OH_ dominates the surface at lower potentials, for $\theta \left({\mathrm{k̂}}^{\exp},u^{eq}\right)$ , the surface remains fully covered by OH at high potentials, while for θ­(**k**_**0**_, *u* ^eq^), a decrease in θ_OH_ with a parallel increase in θ_OOH_ starts around 1.6 V vs RHE. This can be explained by the relatively small value that was estimated for ${\mathrm{k̂}}_{f2}$ (see Table 2), which inhibits the formation of θ_O_ from θ_OH_. This comparison shows that the estimation of rate constants gives direct access to surface coverages. This is insight that cannot be obtained by experiments and can contribute significantly to identifying the limitations at the interface. In addition, the results also illustrate how large the impact of the determined rate constants is on the surface coverages, as the estimated rate constants, ${\mathrm{k̂}}^{\exp}$ , yield significantly different coverages compared to the DFT-derived (predefined) rate constants, **k**_**0**_. These two approaches for obtaining the rate constants and, in extension, the coverages are fundamentally different. Given the apparent large sensitivity of the coverages to the rate constant values, using two distinct approaches to obtain coverages is highly valuable. Together, these two approaches allow us to identify general trends in the obtained coverages and provide insight into the uncertainty associated with these coverages.

**Figure 11:**
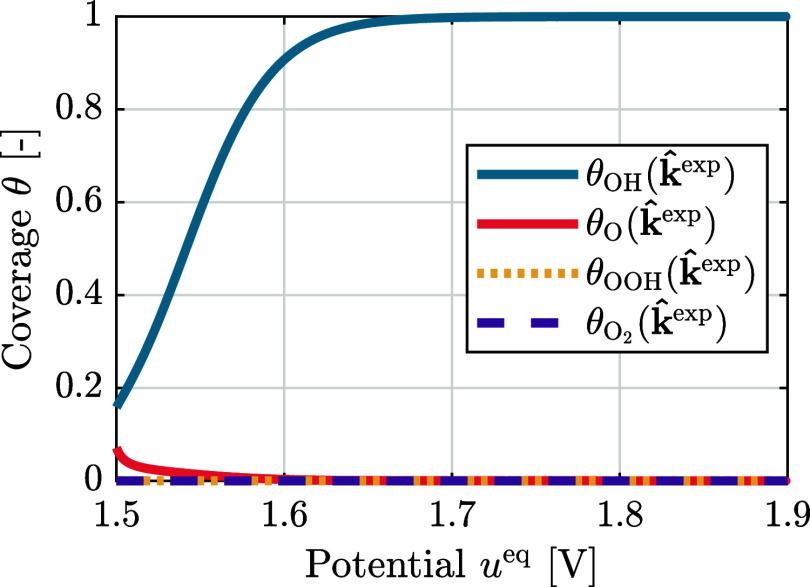
Coverages of the intermediate species, OH, O, OOH, and O_2_, reconstructed from the estimated rate constants, $\theta \left({\mathrm{k̂}}^{\exp},u^{eq}\right)$ , as a function of potential, *u* ^eq^, between 1.5 and 1.9 V vs RHE.

## 5 Conclusions and Recommendations

In this work, reaction rate constants have been estimated from experimental data, providing insight into the electrochemistry at the SEI directly from measurements. The estimation allows for prediction of the measurement data and thus can be used to help identify the limiting processes at the electrochemical interface. The estimation of reaction rate constants from experimental data is an alternative to the calculation of rate constants through first-principles methods, such as DFT, which are limited by computational complexities and costs.

In this work, the rate constants of the oxygen evolution reaction, described by a microkinetic OER model, are estimated from frequency, and potential-dependent EIS measurements. This estimation novelly uses the maximum likelihood estimation to explicitly incorporate measurement uncertainty to improve the rate constant estimation. Notably, our approach also employs for the first time impedance measurements at multiple potentials to estimate a single set of potential-independent rate constants. This work has led to four main outcomes:

1. The approach to estimate the rate constants introduced in this study is capable of accurately estimating the rate constants from synthetic EIS measurements, with the exception of the rate constant *k*_b3_; this rate constant had a relatively high estimation error and is unidentifiable in the used frequency range. Despite this inaccuracy, the surface coverages of intermediate species could be accurately reconstructed from the estimated rate constants.
2. Rate constants were estimated from experimental EIS measurements in a photoelectrochemical setup with a hematite Fe_3_O_2_ anode. However, the accuracy of this estimation was limited by a dominant nonideal capacitive behavior in the measurement data, represented by the CPE exponent α = 0.89 and CPE pseudocapacitance *Q* in equivalent circuit fitting of the experimental data. This behavior is a complex combination of processes that could in part be linked to transport (for fully dominant diffusive transport, α = 0.5). Hence, future work should be focused on improving the microkinetic modeling framework with a detailed description of the transport phenomena. In fact, ongoing efforts by the authors are directed toward incorporating the transport of charge carriers in the semiconductor into the OER model in the present work, which will provide a thorough description of charge carrier diffusion. Such an extended model should in the future be used for the estimation of rate constants. Furthermore, the model used in this work simulates the OER only in the dark. A description of the illumination of the electrode is also implemented in the extended model under development.
3. This study used analysis of the Cramer-Rao lower bound (CRLB) to show that EIS measurements at low frequency and high potential are favorable for the estimation of the rate constants. At low frequency, due to the high impedance of the constant phase element (CPE) compared to *Z*_SEI_, the cell impedance is more sensitive to variations in the rate constants. At high potentials, the overall rate of reactions increases, which also increases the sensitivity to the rate constants.
4. This study highlights the shortcomings of EIS software concerning the manner in which impedance data is exported. The EIS software used in this work only provides the impedance measurements, internally calculated from the potential excitation and current density signals. These impedance measurements lead to systematic errors in the data processing that is required for the estimation of the rate constants. Instead, the individual potential excitation and current density signals should be used in the future to estimate the rate constants most accurately. It has to be confirmed which potentiostat supplier provides these signals individually.

In conclusion, this study demonstrates how combining potential- and frequency-dependent EIS measurements with microkinetic modeling enables the estimation of reaction rate constants and intermediate species coverages without relying on idealized atomistic configurations, aiding in identifying reaction mechanisms directly from measurements. This is a new approach for evaluating and analyzing impedance data in the field of electrochemistry. With the possibility of determining the values of the reaction rate constants, it becomes feasible to directly relate experimental impedance data to electrochemical quantities. These quantities enable modeling or predicting of electrochemical data of different material systems while reducing the number of experimental measurements; this prospect marks great progress in the field.

## Supplementary Material



## Notes

Symbols and parameters used in the simulations, evolutionary strategy algorithm settings, numerical background information, impedance estimator bias, and synthetic estimation, including CPE coefficients (PDF)
