# Supplementary material for: Estimating Reaction Rate Constants from Impedance Spectra: Combining Microkinetic Modeling and Experiments of the Oxygen Evolution Reaction
Source: J Phys Chem C Nanomater Interfaces. 2026 Apr 13;130(16):5781–93. doi: 10.1021/acs.jpcc.6c00968 (PMC13112350; doi:10.1021/acs.jpcc.6c00968)
Supplement: Supplementary file 1 [file jp6c00968_si_001.pdf]

Supporting Information: Estimating Reaction Rate Constants from Impedance Spectra: Combining Microkinetic Modeling and Experiments of the Oxygen Evolution Reaction

B.F.H. van den Boorn,[*,†,‡] F. Vandeputte,[¶] M. van Berkel,[*,§,‡] C. Mempin,[†] D. Sarkar,[†] J. Lataire,[¶] G. Vandersteen,[¶] and A. Bieberle-Hütter[*,†]

†*Electrochemical Materials and Interfaces, DIFFER - Dutch Institute for Fundamental Energy Research, Eindhoven, The Netherlands*

‡*Electrical Engineering, Eindhoven University of Technology, Eindhoven, The Netherlands*

¶*ELEC, Vrije Universiteit Brussel, Brussels, Belgium*

§*Energy Systems and Control, DIFFER - Dutch Institute for Fundamental Energy Research, Eindhoven, The Netherlands*

E-mail: b.f.h.vandenboorn@differ.nl; m.vanberkel@differ.nl; a.bieberle@differ.nl

Phone: +31 (0)40 3334 801

Additional modeling parameters

Symbols and parameters used in the simulations

Table S1: List of symbols and parameters used in the oxygen evolution reaction model for a hematite Fe_2O_3 semiconductor.

Symbol	Parameter	Value	References
k_{max}	VB max rate constant [$\mathrm{cm^4\,s^{-1}}$]	1×10^{-16}	S1
N_{v}	VB density of states [$\mathrm{cm^{-3}}$]	1×10^{22}	S2
N_{c}	CB density of states [$\mathrm{cm^{-3}}$]	4×10^{22}	S2
E_{v}	VB energy level [eV]	2.4	S3
E_{c}	CB energy level [eV]	0.3	S3
λ	Solvent reorganization energy [eV]	1	S4
C_{H}	Helmholtz capacitance [$\mathrm{F\,cm^{-2}}$]	20×10^{-6}	S5
C_{sc}	Space-charge capacitance [$\mathrm{F\,cm^{-2}}$]	-	-
R_{s}	Series resistance [Ω]	-	-
T	Temperature [K]	298	-
ϵ_0	permittivity of free space [$\mathrm{F\,cm^{-1}}$]	8.85×10^{-14}	-
ϵ_{r}	relative permittivity of the Fe_2O_3 semiconductor [-]	38.2	S6
N_{D}	doping density [$\mathrm{cm^{-3}}$]	4×10^{19}	
pH	- [-]	13.8	-
xOH	mole fraction of hydroxide ions (from pH) [-]	1.12×10^{-2}	-
xH_2O	mole fraction of water (from pH) [-]	9.89×10^{-1}	-
ΔG_1	Gibbs free energy of OER step 1 [eV]	1.4610	S7
ΔG_2	Gibbs free energy of OER step 2 [eV]	2.0155	S7
ΔG_3	Gibbs free energy of OER step 3 [eV]	1.2042	S7
ΔG_4	Gibbs free energy of OER step 4 [eV]	0.2393	S7

Evolutionary strategy algorithm settings

The Evolutionary strategy (ES) algorithm that is used to perform an initial optimization of the rate constants is implemented in the companion paper.[S8] The obtained rate constants $\hat{k}_{\mathrm{ES}}$ are used as initial guesses for the Levenbergh-Marquardt algorithm. The ES algorithm is implemented with bound constraints, where the estimate of the rate constants from the experimental data is found between $[1 \times 10^{-30}, 1 \times 10^{-9}]$ $\mathrm{cm^4\,s^{-1}}$ and for the pseudocapacitances between $[1 \times 10^{-7}, 1 \times 10^{-3}]$ $\mathrm{F\,cm^{-2}\,s^{-0.2}}$. The options in Table S2 have been set according to the strategy in.[S8]

Table S2: Evolutionary Strategy (ES) Algorithm Options.

ES option	Value
Parent Population Size	10 / 20
Offspring Population Size	100
Selection Strategy	,
Recombination method	Discrete
Maximum Number of Generations	8000

Numerical background information

System sensitivity and conditioning

The sensitivity of the output to changes in the input or round-off errors can be represented by the condition number for inversion κ. The condition number is defined as the ratio of the largest $\sigma_{\max}$ and smallest $\sigma_{\min}$ singular values of system matrix $\boldsymbol{A}$

$$\kappa(\boldsymbol{A}) = \frac{\sigma_{\max}}{\sigma_{\min}}, \tag{1}$$

where $\sigma_{\max} \geq \cdots \geq \sigma_{\min} \geq 0$ represent the singular values of $\boldsymbol{A}$. A condition number close to 1 $\kappa \approx 1$ means good conditioning, while a large condition number $\gg 1$ means that the system is ill conditioned. Figure S1 shows the condition number over potential of the linearized OER model, revealing that the model is especially ill-conditioned at low potentials. However, the conditioning improves for larger potentials, where the condition number decreases at an exponential rate.

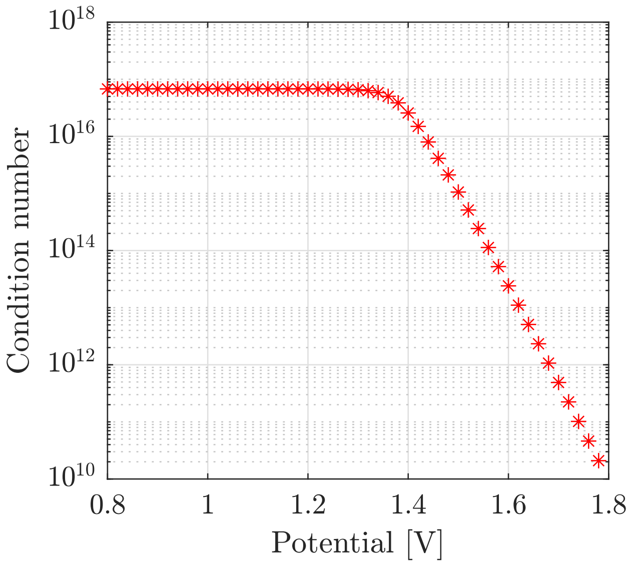

Figure S1: Condition number of matrix A of the linearized system at potentials $V_{\mathrm{app}} = [0.8, 1.8]$

Impedance estimator bias

The impedance estimator calculated using the 'simple approach' exhibits a systematic error, or bias, compared to the noiseless impedance value. This apparent bias can be explained by expanding the data $U(M)$ and $J(M)$ (the dependency on f and ρ is dropped) into the noiseless term and the noise, for both the 'simple approach' (SA) estimator and the 'errors-in-variables' (EV) estimator.[S9] The asymptotic value of the estimators is obtained by taking the limit for $M \to \infty$,

$$\begin{aligned}\lim_{M\to\infty} \hat{Z}_{\mathrm{ev}}(M) &= \frac{\lim_{M\to\infty} \frac{1}{M}\sum_{m=1}^{M}(U_0 + N_{\mathrm{U}}^{[m]})}{\lim_{M\to\infty} \frac{1}{M}\sum_{m=1}^{M}(J_0 + N_{\mathrm{J}}^{[m]})} \\ &= \frac{U_0}{J_0} = Z_0,\end{aligned} \tag{2}$$

$$\begin{aligned}\lim_{M\to\infty} \hat{Z}_{\mathrm{sa}}(M) &= \lim_{M\to\infty} \frac{1}{M}\sum_{m=1}^{M}\frac{U_0 + N_{\mathrm{U}}^{[m]}}{J_0 + N_{\mathrm{J}}^{[m]}} \\ &= Z_0\left(1 + \frac{\sigma_{\mathrm{J}}^2}{J_0^2}\right),\end{aligned} \tag{3}$$

where σ_{J}^2 is defined as the sum of squared current density noise distributions, which converges to a constant value that is not equal to zero, as the noise in non-circular complex in nature,[S9]

$$\lim_{M\to\infty} \frac{1}{M} \sum_{m=1}^{M} \left(N_{\mathrm{J}}^{[m]}\right)^2 = \sigma_{\mathrm{J}}^2 \neq 0. \tag{4}$$

It is evident that the estimator $\hat{Z}_{\mathrm{ev}}(M)$ converges to the noiseless value Z_0 for increasing M, whereas the estimator $\hat{Z}_{\mathrm{sa}}(M)$ does not. The bias is equal to the term $\frac{\sigma_{\mathrm{J}}^2}{J_0^2}$, but without the current density measurements $J^{[m]}$, it cannot be calculated or approximated. It can be noted that, for higher SNR, the bias is smaller, and this decreases the extent to which the estimation is affected. However, to avoid bias altogether, both the potential $U^{[m]}$ and the current $J^{[m]}$ should be provided by the software of the EIS potentiostat.

Supporting simulations and experiments

Synthetic estimation including CPE coefficients

This section presents the results of a single estimation of the rate constants $\hat{\mathbf{k}}_{\mathrm{sim}}$ (Table S3), CPE pseudocapacitances $\hat{\mathbf{Q}}_{\mathrm{cmb}}^{\mathrm{sim}}$ (Table S4) and CPE exponents $\hat{\alpha}$ (Table S5). The maximum percentage relative error ϵ_{k} is found for k_{b3}.

Table S3: Predefined rate constants $\mathbf{k}_0$ and estimated rate constants $\hat{\mathbf{k}}$, and the percentage relative error ϵ_{k}.

	$\mathbf{k}_0$	$\hat{\mathbf{k}}^{\mathrm{sim}}$	ϵ_{k} [%]
k_{f1}	4.69×10^{-17}	4.70×10^{-17}	2.12×10^{-1}
k_{b1}	9.80×10^{-28}	9.80×10^{-28}	3.38×10^{-2}
k_{f2}	4.40×10^{-20}	4.40×10^{-20}	2.08×10^{-3}
k_{b2}	2.19×10^{-21}	2.19×10^{-21}	1.45×10^{-2}
k_{f3}	1.56×10^{-16}	1.56×10^{-16}	1.00×10^{-2}
k_{b3}	1.48×10^{-31}	-3.61×10^{-29}	2.45×10^{4}
k_{f4}	1.47×10^{-19}	1.47×10^{-19}	2.61×10^{-2}

Table S4: Predefined CPE pseudocapacitances $\mathbf{Q}_{\mathrm{cmb},0}$ and estimated pseudocapacitances $\hat{\mathbf{Q}}_{\mathrm{cmb}}$, and the percentage relative error ϵ_{Q}.

	$\mathbf{Q}_{\mathrm{cmb},0}$	$\hat{\mathbf{Q}}_{\mathrm{cmb}}^{\mathrm{sim}}$	ϵ_{Q} [%]
$Q_{\mathbf{cmb},1.5V}$	6.00×10^{-6}	6.00×10^{-6}	5.01×10^{-3}
$Q_{\mathbf{cmb},1.6V}$	5.86×10^{-6}	5.86×10^{-6}	3.05×10^{-3}
$Q_{\mathbf{cmb},1.7V}$	5.74×10^{-6}	5.74×10^{-6}	1.19×10^{-3}
$Q_{\mathbf{cmb},1.8V}$	5.65×10^{-6}	5.65×10^{-6}	1.55×10^{-2}

Table S5: Predefined CPE exponents α_0 and estimated exponents $\hat{\alpha}$, and the percentage relative error ϵ_{α}.

	α_0	$\hat{\alpha}^{\mathrm{sim}}$	ϵ_{α} [%]
$\alpha_{1.5V}$	0.70	0.70	5.55×10^{-4}
$\alpha_{1.6V}$	0.70	0.70	5.62×10^{-4}
$\alpha_{1.7V}$	0.70	0.70	2.89×10^{-4}
$\alpha_{1.8V}$	0.70	0.70	1.95×10^{-3}

Series resistance and pseudocapacitance of experimental estimation

The series resistance R_{s} is extracted from the measurements as the impedance value at the highest measured frequency. The values of R_{s} are given in Figure S2a. The estimated pseudocapacitances of CPE $\hat{Q}(u_{\rho}^{\mathrm{eq}})$ are shown in Figure S2b.

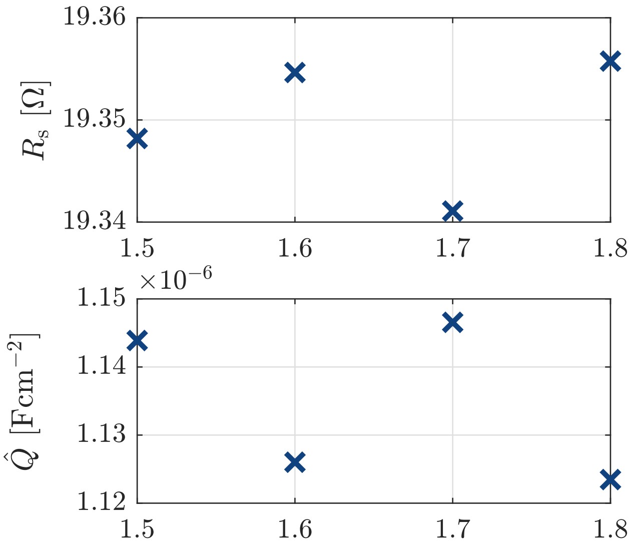

Figure S2: From the experimental EIS measurements at each potential, a) the series resistances R_{s} are extracted, and b) the CPE prefactors $\hat{Q}(u_{\rho}^{\mathrm{eq}})$ are estimated.

References

(S1) Lewis, N. S. Progress in Understanding Electron-Transfer Reactions at Semiconductor/Liquid Interfaces. *The Journal of Physical Chemistry B* **1998**, *102*, 4843–4855.

(S2) Cendula, P.; Tilley, S. D.; Gimenez, S.; Bisquert, J.; Schmid, M.; Grätzel, M.; Schumacher, J. O. Calculation of the Energy Band Diagram of a Photoelectrochemical Water Splitting Cell. *The Journal of Physical Chemistry C* **2014**, *118*, 29599–29607.

(S3) Barroso, M.; Pendlebury, S. R.; Cowan, A. J.; Durrant, J. R. Charge carrier trapping, recombination and transfer in hematite (α-Fe2O3) water splitting photoanodes. *Chem. Sci.* **2013**, *4*, 2724–2734.

(S4) George, K.; van Berkel, M.; Zhang, X.; Sinha, R.; Bieberle-Hütter, A. Impedance Spectra and Surface Coverages Simulated Directly from the Electrochemical Reaction Mechanism: A Nonlinear State-Space Approach. *J. Phys. Chem. C* **2019**, *123*, 9981–9992.

(S5) Hankin, A.; Bedoya-Lora, F. E.; Alexander, J. C.; Regoutz, A.; Kelsall, G. H. Flat band potential determination: avoiding the pitfalls. *J. Mater. Chem. A* **2019**, *7*, 26162–26176.

(S6) George, K.; Khachatrjan, T.; van Berkel, M.; Sinha, V.; Bieberle-Hütter, A. Understanding the Impact of Different Types of Surface States on Photoelectrochemical Water Oxidation: A Microkinetic Modeling Approach. *ACS Catal.* **2020**, *10*, 14649–14660.

(S7) Zhang, X.; Klaver, P.; van Santen, R.; van de Sanden, M. C. M.; Bieberle-Hütter, A. Oxygen Evolution at Hematite Surfaces: The Impact of Structure and Oxygen Vacancies on Lowering the Overpotential. *The Journal of Physical Chemistry C* **2016**, *120*, 18201–18208.

(S8) Vandeputte, F.; van den Boorn, B. F. H.; van Berkel, M.; Bieberle-Hütter, A.; Vandersteen, G.; Lataire, J. Estimating Reaction Rate Constants from Impedance Spectra:

Simulating the Multistep Oxygen Evolution Reaction. *Electrochimica Acta* **2026**, *559*, 148667.

(S9) Pintelon, R.; Schoukens, J. *System Identification: A frequency Domain Approach*, 2nd ed.; Wiley / IEEE Press, 2012; IEEE Press, Wiley.
